# Enhanced Interfacial Adhesion and Dynamic Seawater Durability of Basalt Fiber/Epoxy Composites via PDA-Assisted In Situ Growth of UiO-66-NH_2_

**DOI:** 10.3390/polym18192425

**Published:** 2026-10-05

**Authors:** Yingquan Zhou, Guoqian Xie, Bin Wang, Chunxia Zhao, Hui Li, Dong Xiang

**Affiliations:** 1 School of New Energy and Materials, Southwest Petroleum University, Chengdu 610500, China; 18384914476@163.com (Y.Z.); 18331953657@163.com (G.X.); binwang2008@swpu.edu.cn (B.W.); polychem@swpu.edu.cn (C.Z.); lihui@swpu.edu.cn (H.L.); 2 Sichuan Engineering Technology Research Center of Basalt Fiber Composites Development and Application, Southwest Petroleum University, Chengdu 610500, China

**Keywords:** basalt fiber-reinforced composites, interface modification, UiO-66-NH_2_, marine durability, seawater corrosion resistance

## Abstract

Durable basalt fiber-reinforced polymer (BFRP) composites are needed for marine and offshore structures, where seawater exposure and hydrodynamic shear progressively degrade the fiber–matrix interface. To address this challenge, a hierarchical polydopamine (PDA)/UiO-66-NH_2_ interphase was constructed on basalt fibers through PDA-assisted in situ growth. PDA served as an adhesive interlayer and nucleation platform, enabling dense anchoring of UiO-66-NH_2_ crystals. Compared with that of unmodified fibers, the optimized interface increased single-fiber tensile strength by 23.9% and interfacial shear strength by 67.9%. At the laminate level, flexural strength and interlaminar shear strength increased by 43.5% and 44.6%, respectively, accompanied by a transition from interfacial debonding to predominantly resin cohesive failure. Under dynamic simulated-seawater exposure, the modified composites showed lower mass change and improved mechanical property retention. The enhanced performance is attributed to improved interfacial interactions, micro-/nanoscale mechanical interlocking, and delayed ingress of corrosive species. This work provides an interface engineering strategy for mechanically robust and seawater-durable BFRP composites.

## 1. Introduction

Basalt fiber-reinforced polymer (BFRP) composites are promising for load-bearing marine and offshore components, including coastal infrastructure, marine pipelines, and tidal energy structures, where materials must withstand seawater exposure, mechanical loading, and hydrodynamic shear. Compared with conventional glass fiber composites, basalt fibers offer favorable mechanical and chemical stability, whereas they provide a more cost-effective and electrically insulating alternative to carbon fibers [1,2]. Nevertheless, the long-term reliability of BFRP is governed by the fiber–matrix interphase. Seawater uptake and aggressive ions can weaken interfacial load transfer, and flowing water further accelerates this degradation through shear-assisted damage [1,2,3].

Epoxy resins are widely used as matrices for marine composites because of their adhesion, low curing shrinkage, and processing versatility. However, moisture absorption can plasticize the epoxy network and facilitate ion transport along interfacial defects, eventually promoting fiber–matrix debonding [4,5]. Recent studies have shown that BF surface engineering can improve interfacial performance: plasma activation enhances BF/epoxy adhesion [6], hydrophobic modification improves the durability of BFRP bars [3], and bioactive cross-scale functionalization improves seawater corrosion resistance [4]. These studies establish the value of interface design, but most focus on either initial adhesion or durability under relatively conventional exposure conditions. Maintaining both efficient stress transfer and interfacial stability under coupled seawater attack and hydrodynamic shear remains insufficiently understood.

Polydopamine (PDA) is a useful platform for addressing this challenge because it forms adherent coatings on diverse substrates and supplies catechol- and amine-containing sites for secondary functionalization [7,8,9]. PDA-based treatments have improved fiber–polymer interactions, including in basalt fiber composites [10]. However, a PDA layer alone may have limited stiffness and barrier capability during prolonged marine exposure. Incorporating a rigid secondary phase is therefore necessary to construct an interface that combines adhesion, reinforcement, and resistance to seawater ingress.

Zr-based UiO-66-NH_2_ is particularly attractive because of its high aqueous stability and amino-functionalized framework, which can promote compatibility with epoxy networks [11,12,13]. PDA can also act as a substrate-bound nucleation platform for in situ MOF growth [14,15]. Although MOF/PDA hybrid interfaces have improved the mechanical performance of carbon fiber composites [16], their application to chemically inert basalt fibers and their durability under flowing seawater remain largely unexplored [17,18]. In particular, the relationships between a hierarchical PDA/UiO-66-NH_2_ interphase, multiscale load transfer, and dynamic seawater degradation have not been established [19,20].

Motivated by these gaps, this study develops a PDA-assisted in situ UiO-66-NH_2_ growth strategy on basalt fibers. The novelty lies in three aspects: (i) PDA serves simultaneously as an adhesive bridge and a nucleation platform to immobilize UiO-66-NH2 on BF; (ii) the resulting interphase is correlated with fiber strength, interfacial shear strength, and laminate mechanical properties to clarify its multiscale strengthening role; and (iii) its durability is assessed under dynamic simulated-seawater exposure with controlled hydrodynamic shear. This work therefore provides a structure–interface–durability framework for designing mechanically robust and seawater-durable BFRP composites.

## 2. Experiments

### 2.1. Materials

Chopped basalt fibers (BFCS615-10-3; Sichuan Qianyi) and plain-woven basalt fabric and prepregs (Yixing Star Carbon Fiber Composites Co., Ltd., Yixing City, Wuxi, Jiangsu Province, China) were used. The fabric had a nominal areal density of 300 ± 15 g/m^2^ (300.4 ± 6.2 g/m^2^; *n* = 5, 100 mm × 100 mm specimens), warp/weft densities of 6 ends cm^−1^ × 6 picks cm^−1^, a filament diameter of 13 ± 2 µm, and a sizing content of 0.8 ± 0.1 wt% (Figure S1); the prepreg epoxy content was 42 wt%. Dopamine hydrochloride (98%), 2-aminoterephthalic acid (NH2-BDC, 98%), ZrCl_4_ (98%), acetic acid (99.5%), Tris (hydroxymethyl)aminomethane (99.9%), and N,N-dimethylbenzylamine accelerator were supplied by Macklin Reagents (Shanghai, China). Ethanol (99.7%), acetone (99.5%), and DMF (99.5%) were obtained from Chengdu Kelong Chemical Co., Ltd. (Chengdu, China); E51 epoxy resin (epoxy value 0.51 mol/100 g) and MTHPA curing agent (>98%) were obtained from Nantong Xingchen Synthetic Material Co., Ltd. (Nantong City, Jiangsu Province, China) and Shanghai Macklin Biochemical Co., Ltd. (Fengxian District, Shanghai, China), respectively. All reagents were used as received; deionized water (18.2 MΩ cm) was used throughout.

### 2.2. Sample Preparation

**Figure 1:**
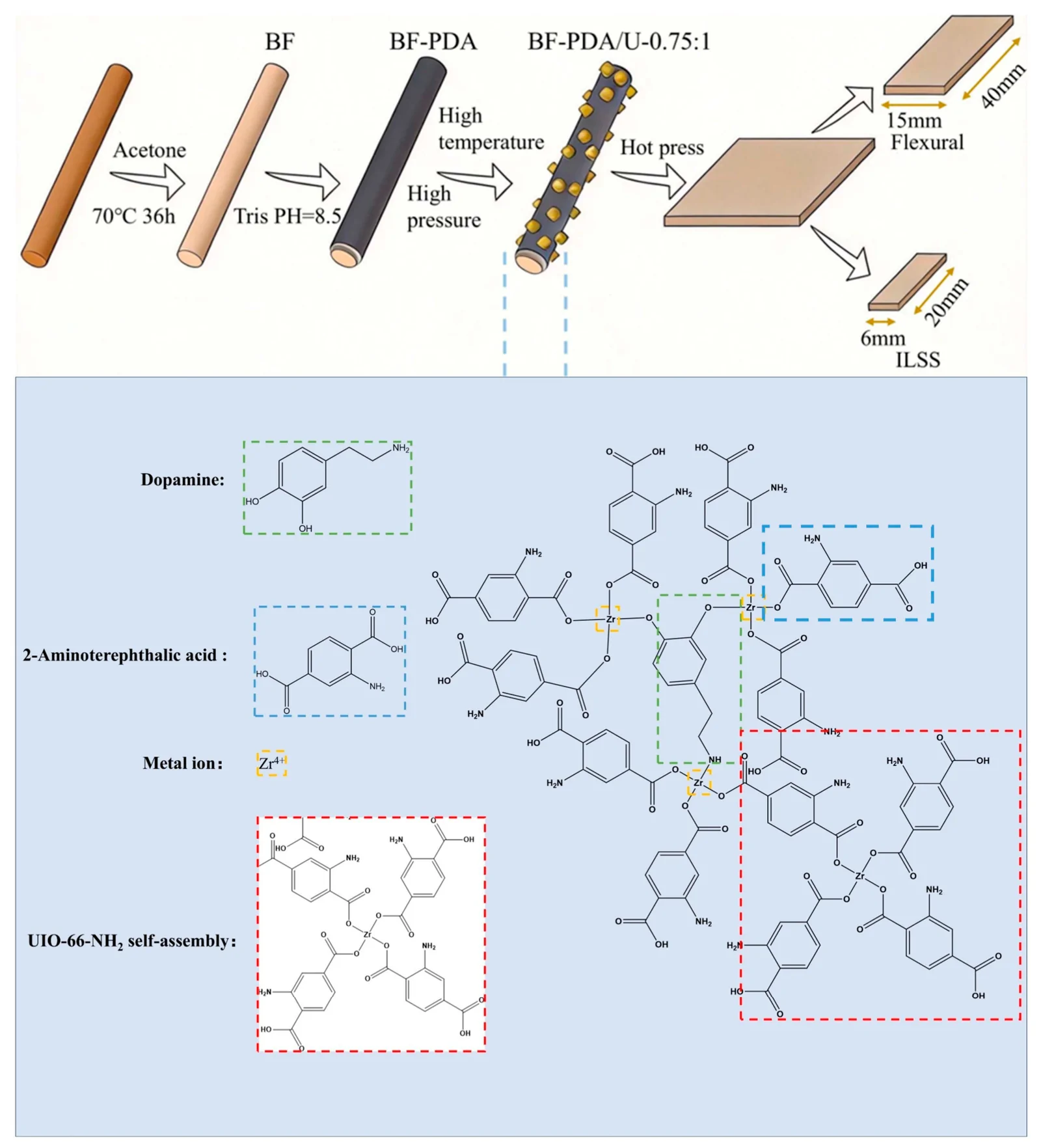
Sample preparation and mechanism diagram.

**Figure 2:**
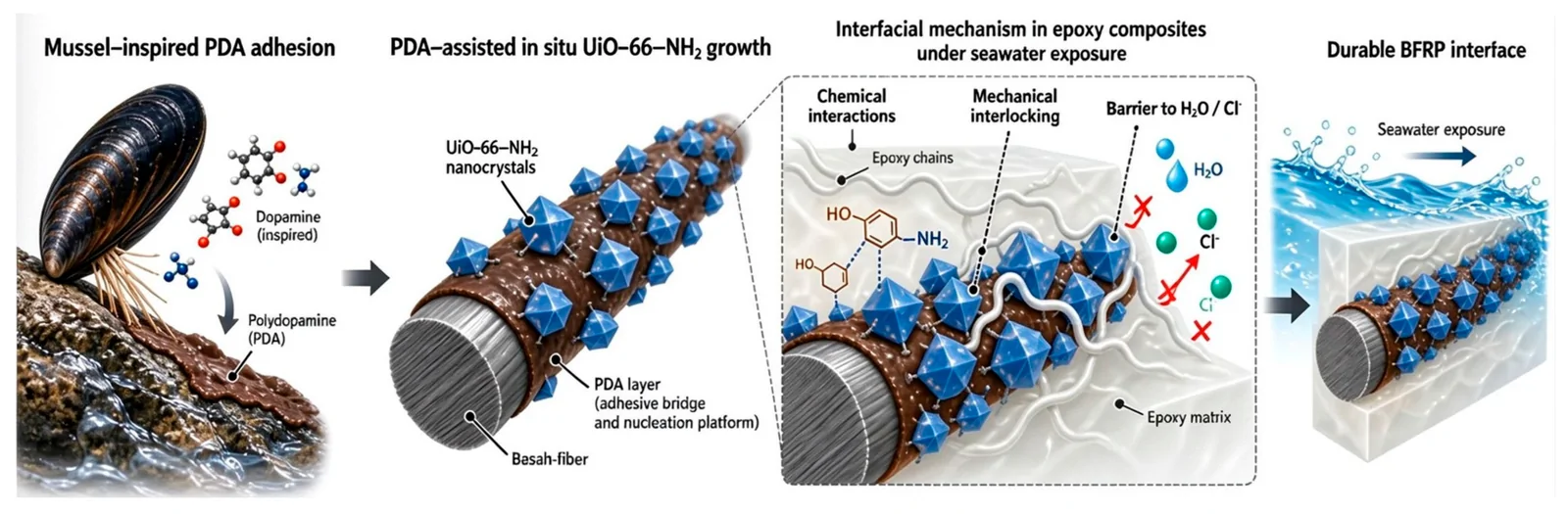
Schematic diagram of modification mechanism.

Raw BF was desized in acetone at 70 °C for 36 h, rinsed sequentially with ethanol and deionized water, and dried at 60 °C. For PDA deposition, dopamine hydrochloride (2 g L^−1^) was freshly dissolved in 10 mM Tris buffer (pH 8.5, adjusted with 1 M HCl). Fibers were immersed at 25 ± 1 °C under magnetic stirring (300 rpm) in air for 12–48 h to obtain BF-PDA. For in situ UiO-66-NH2 growth, ZrCl_4_ and NH_2_-BDC were dissolved in 60 mL DMF at molar ratios of 0.5:1, 0.75:1, 1:1, 1:0.75, and 1:0.5 (total precursor concentration, 30 mM), with acetic acid (2 wt% of precursor solution) as a modulator. BF-PDA was placed in a Teflon-lined autoclave, heated at 2 °C min^−1^ to 120 °C, held for 24 h, and naturally cooled. The fibers were washed three times with fresh DMF and ethanol and vacuum-dried at 60 °C for 12 h. Samples are designated BF-PDA/U-x:y, where x:y is the ZrCl_4_:NH_2_-BDC molar ratio. BF-PDA/U-0.75:1 prepared with 36 h PDA deposition was used in subsequent experiments. The modification route is summarized in Figure 1, and the proposed interfacial mechanism is illustrated in Figure 2.

Epoxy resin (E51) and MTHPA were mixed at a 10:8 mass ratio and preheated at 70 °C for 10 min solely to adjust the viscosity. The mixture was hand-laid onto modified BF fabric in a release-agent-treated mold. Eight plies were used to obtain a nominal fiber volume fraction of 45 ± 2% (matrix burn-off, ASTM D3171-99-(2004)) [21]. Demolding cloth and polyimide release films were placed on both sides before hot-pressing at 120 °C and 10 MPa for 3 h. The resulting laminates (180 mm × 30 mm × 2 mm) had a thickness tolerance of ±0.1 mm, determined from five random micrometer measurements, and were cut into flexural and short-beam ILSS specimens. The fabrication sequence is shown in Figure 1.

### 2.3. Dynamic Corrosion

**Figure 3:**
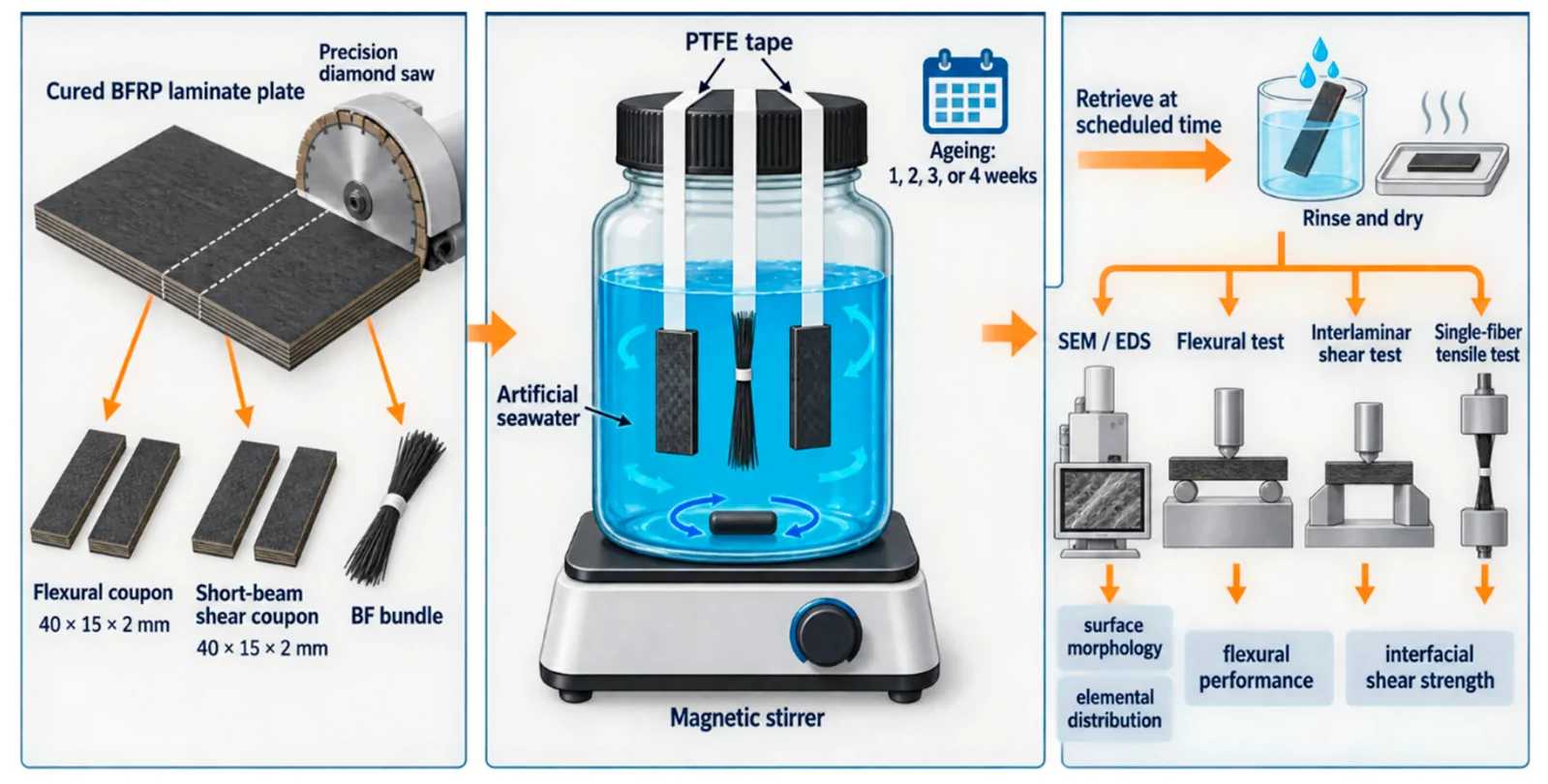
Experimental workflow for dynamic seawater ageing and post-exposure characterization. Note: Generative AI tool (Doubao-Seed-2.1, ByteDance, Beijing, China) was used for auxiliary visual rendering of Figure 2 and Figure 3. Authors defined all scientific logic, structural layout and core elements of these figures. AI only provided preliminary sketch rendering output. All figures were manually revised, proof-read and confirmed by authors to match real experimental mechanism and workflow. GenAI was not used for experimental design, data generation, data analysis, result interpretation or text drafting.

Pristine and modified BF and their corresponding composites were exposed to dynamic simulated seawater prepared based on ASTM D1141-98 (2013) [22] (Table 1; salinity ≈ 3.5 wt%, pH 8.2 adjusted with 0.1 M NaOH). Flexural and short-beam coupons together with BF bundles were suspended in wide-mouth bottles using PTFE tape secured by the bottle cap and immersed in the simulated seawater (Figure 3). The bottles were placed on magnetic stirrers, with a magnetic bar at the bottom. The stirring speeds were 0, 600, 1200, and 1800 rpm. Specimens were retrieved after 1, 2, 3, and 4 weeks; the solution was renewed every 7 days.

### 2.4. Characterization

The surface/fracture morphology and elemental distributions were characterized by FE-SEM/EDS (ZEISS EVO MA15, 15 kV; Oxford X-Max 20, Carl Zeiss Microscopy GmbH, Jena, Germany). Samples were mounted on conductive tape and Au-sputtered (Quorum Q150R S, 20 mA, 60 s, Ted Pella Inc. Redding, CA, USA). Corroded samples were imaged at a working distance of 8.5 mm and magnifications of ×500 and ×20,000; at least six filaments per condition were examined. EDS was conducted on fiber surfaces and on filaments used for tensile tests. Surface chemistry was analyzed by XPS (Nexsa, Thermo Scientific, Thermo Fisher Scientific, Cambridge, UK; Al Kα, 1486.6 eV; 400 µm spot; 50 eV survey and 20 eV high-resolution pass energies; C 1s = 284.8 eV), ATR-FTIR (Nicolet 6700, Thermo Fisher Scientific, Madison, WI, USA; 4000–400 cm^−1^, 4 cm^−1^, 32 scans), and Raman spectroscopy (B&W TEK BWS465-785S, Thermo Scientific, Madison, WI, USA; 785 nm, 200–2000 cm^−1^). The crystal phase and surface roughness were determined by XRD (Bruker D8 Advance, PANalytical Almelo Netherlands; Cu Kα, λ = 1.5406 Å; 5–50°, 2° min^−1^) and AFM (Bruker Dimension Icon, Bruker Nano GmbH, Karlsruhe, Germany; tapping mode; 5 µm × 5 µm). The coating mass fraction and semi-quantitative PDA content were assessed by SEM-EDS (Carl Zeiss Microscopy GmbH, Jena, Germany). The test workflow is shown in Figure 3.

Static contact angles of E51/MTHPA and deionized water were measured by the sessile drop method (KRUSS DSA25) using at least eight droplets on five filaments per group. IFSS was determined by single-fiber microdroplet debonding using a 50 µm epoxy droplet cured at 120 °C for 3 h, following the Kelly–Tyson approach; debonded surfaces were examined by SEM. Tensile and bending tests were performed on a CMT4104 universal testing machine (MTS Systems, Lanzhou Zhongke Kaihua Technology Development Co., Ltd. Lanzhou, China; 10 kN load cell), and load–displacement or stress–strain curves were recorded at 20 Hz (Figure 12). Test procedures and specimen dimensions are shown in Figure 3.

Single-fiber properties before and after modification were measured in accordance with ASTM C1557-2014 [23] using a YG001A tester (Wenzhou Jigao Testing Instrument Co., Ltd., Wenzhou, China; *n* = 50, mean reported). The gauge length and tensile rate were 20 mm and 10 mm min^−1^, respectively. Tensile strength was calculated as σ_t_ = F_u_/(πr^2^), where F_u_ is the breaking force and r is the filament radius measured from SEM fracture images. Representative stress–strain curves were used to determine the tensile modulus (Figure 10).

The mass change of 40 mm × 15 mm × 2 mm composites was determined gravimetrically (Shanghai Sunny Hengping Scientific Instrument Co., Ltd., Shanghai, China; 0.1 mg; *n* = 5) before exposure and after 1, 2, 3, and 4 weeks. Before each measurement, specimens were oven-dried at 60 °C for 24 h and cooled in a desiccator. Mass change was calculated as M_t_ = (m_t_ − m_0_)/m_0_ × 100%, where m_0_ and m_t_ are the oven-dry masses before and after exposure.

Flexural properties were measured according to GB/T 1449-2005 [24] at room temperature using 40 mm × 15 mm × 2 mm specimens (span-to-thickness ratio, 16:1), a 2 mm min^−1^ crosshead rate, and a 10 kN load cell. The flexural strength and modulus were obtained from stress–strain curves; the mean of five valid specimens is reported (Figure 12). ILSS was measured by short-beam testing in accordance with GB/T 1450.1-2005 [25] under the same loading conditions; the mean of five valid results is reported. ILSS was calculated as τ = 0.75F_max_/(bh), where F_max_ is the maximum load before failure and b and h are the specimen width and thickness, respectively.

## 3. Results and Discussion

### 3.1. Surface Morphology and Chemical Analysis of Bf-Pda Before and After Mof Modification

#### 3.1.1. Surface Morphology of Bf-Pda

**Figure 4:**
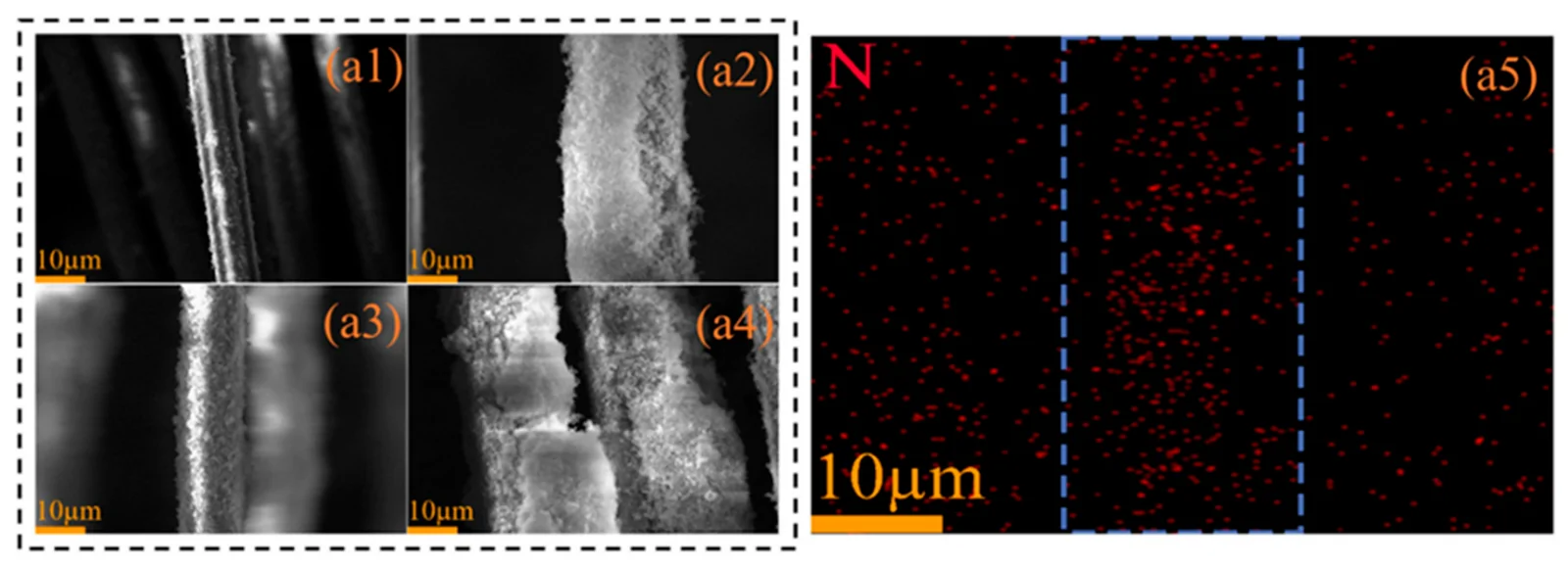
SEM and EDS images of BF-PDA at different polymerization times: (**a1**) 12 h; (**a2**) 24 h; (**a3**) 36 h; (**a4**) 48 h; (**a5**) 36 h.

Figure 4(a1–a4) show the evolution of PDA coverage with the polymerization time. At 12 h, discontinuous island-like deposits were visible; at 24 h, they coalesced into an almost continuous but still heterogeneous film. The 36 h sample showed the most uniform and dense coating, whereas 48 h produced local thickening, roughening and microcracks, suggesting overgrowth. This evolution is consistent with the reported nucleation, deposition and cross-linking of dopamine during PDA formation [8,9,26]. The transition from discontinuous island-shaped deposits at 12 h to a dense uniform film at 36 h demonstrates progressive dopamine polymerization and cross-linking on the basalt fiber surface. A continuous PDA interlayer is critical here: it supplies abundant catechol and amine anchoring sites for the subsequent heterogeneous nucleation of UiO-66-NH_2_ MOF crystals. Over-polymerization at 48 h induces local micro-cracks and uneven thickening, which would act as surface defects and impair the integrity of the final hybrid interphase; therefore, 36 h was selected as the optimal deposition duration.

EDS acquired at five positions per filament (Figure 4(a5), Figures S3 and S4) showed that N increased from 0.0 at% for desized BF to 3.1 ± 0.4 at% for BF-PDA, while Si decreased from 21.8 ± 1.1 to 17.2 ± 0.9 at%; the O/N ratio was 4.6 ± 0.3. The N signal and attenuation of the underlying silicate signal support reproducible PDA coverage [10,26].

#### 3.1.2. Surface Morphology Before and After Mof Modification

**Figure 5:**
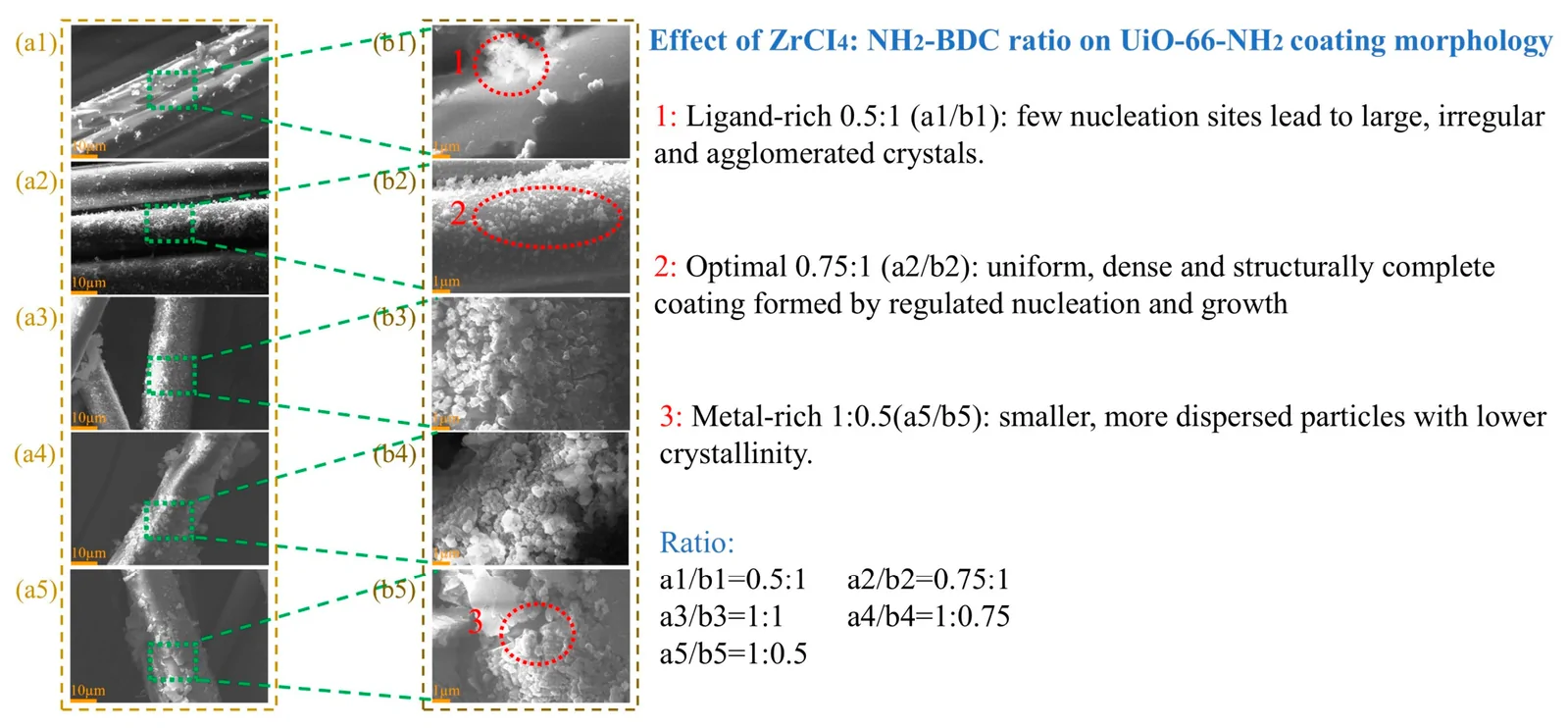
Surface morphology of modified fibers with different molar ratios: (**a1**) 0.5:1; (**a2**) 0.75:1; (**a3**) 1:1; (**a4**) 1:0.75; (**a5**) 1:0.5; (**b1**–**b5**) Corresponding to its local enlarged view.

Figure 5 directly shows that the ZrCl_4_:NH_2_-BDC ratio controlled the particle morphology and the spatial coverage of the BF-PDA surface. Under the ligand-rich 0.5:1 condition (a1/b1), the deposited particles were relatively large, irregular and locally agglomerated. Under the metal-rich 1:0.5 condition (a5/b5), the particles were smaller and more isolated, leaving a less continuous surface decoration. In contrast, the 0.75:1 sample (a2/b2) showed a dense layer of more uniformly distributed particles along the fiber. Thus, the selection of 0.75:1 was based not only on the absence of conspicuous aggregates but also on the more homogeneous coverage apparent in the low- and high-magnification images. The observed ratio-dependent morphology is consistent with the known sensitivity of in situ UiO-66-NH_2_ deposition to the precursor environment, although the present images do not independently resolve deposition kinetics [13].

The ratio-dependent SEM morphology is consistent with a different balance between heterogeneous nucleation and subsequent particle growth: a surface populated by isolated particles or by large clusters is less uniform than one carrying a dense, spatially continuous particle layer. No specific defect or bonding mechanism is inferred from morphology alone. Rather, Figure 5 provides the morphological basis for selecting 0.75:1 as the condition most likely to produce a consistent fiber–resin interphase. PDA-mediated modification of basalt fibers and MOF/PDA hybrid interphases on carbon fibers provide relevant precedents for the present organic–inorganic interface design [10,16].

**Figure 6:**
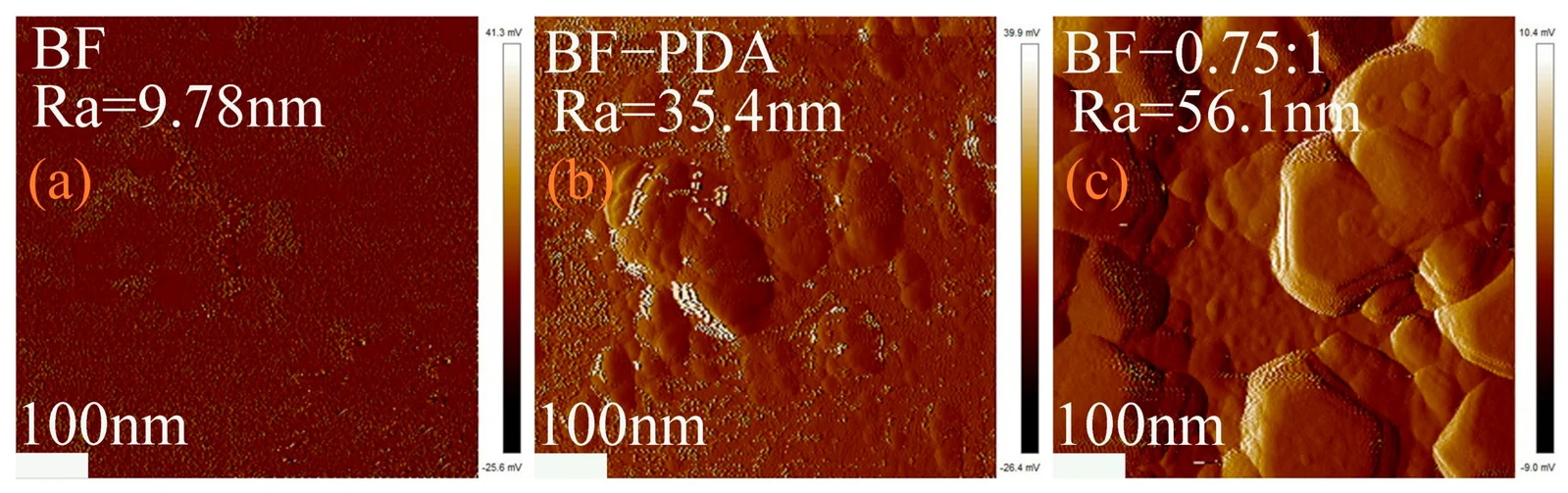
AFM images of (**a**) BF, (**b**) BF-PDA, and (**c**) BF-PDA/U-0.75:1.

**Figure 7:**
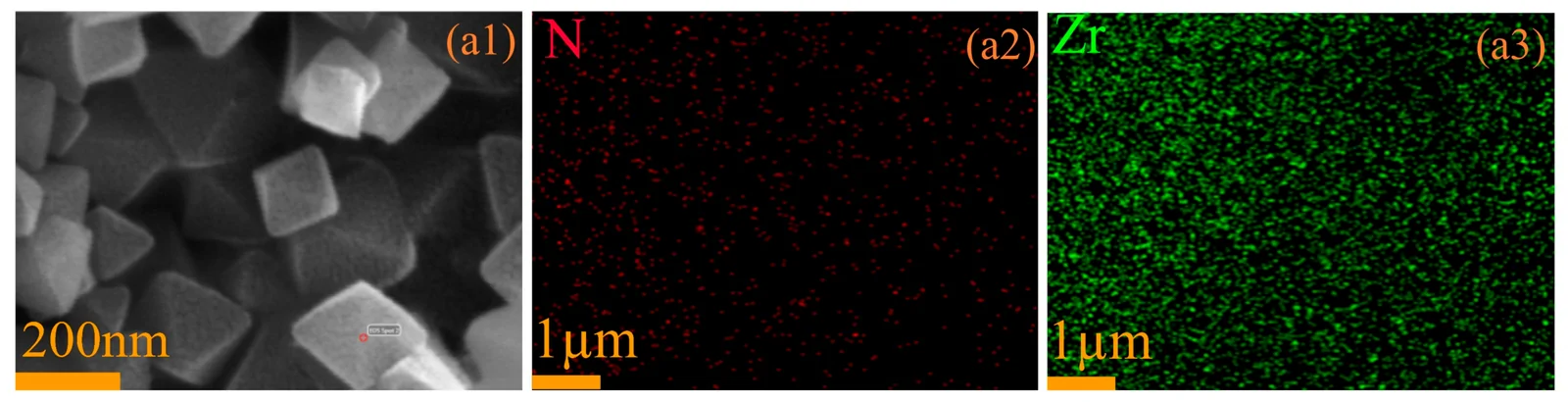
SEM and EDS images of UiO-66-NH_2_, (**a1**) SEM image of UiO-66-NH_2_; (**a2**) Distribution map of N element in the SEM image of UiO-66-NH_2_; (**a3**) Distribution map of Zr element in the SEM image of UiO-66-NH_2_.

AFM (Figure 6) shows a sequential increase in arithmetic surface roughness Ra from pristine BF (9.78 nm) to BF-PDA (35.4 nm) and further to BF-PDA/U-0.75:1 (56.1 nm), quantitatively confirming successful hierarchical coating construction. The raised micro–nanoscale topography contributes to a mechanical interlocking effect when in contact with the epoxy resin matrix, which partially accounts for the improved interfacial shear strength observed in single-fiber tests. The SEM images further reveal that the precursor molar ratio strongly governs MOF crystal size and homogeneity. Excess ligand leads to large agglomerated particles, while a metal-rich condition yields sparse isolated crystals; both scenarios produce discontinuous surface decoration and weaken interphase integrity. Only the 0.75:1 ratio generated densely and homogeneously distributed polyhedral UiO-66-NH_2_ particles (~150–250 nm), building a complete organic–inorganic hybrid interphase. The optimized product showed mainly octahedral/polyhedral particles of approximately 200 nm (150–250 nm) [13,27]. EDS of the coating (Figure 7) detected Zr and N, with homogeneous elemental maps. These observations support UiO-66-NH_2_ loading and explain the greater roughness subsequently correlated with wetting and IFSS (Section 3.2.2) [28,29].

#### 3.1.3. Surface Chemical Analysis of Bf-Pda Before and After Mof Modification

**Figure 8:**
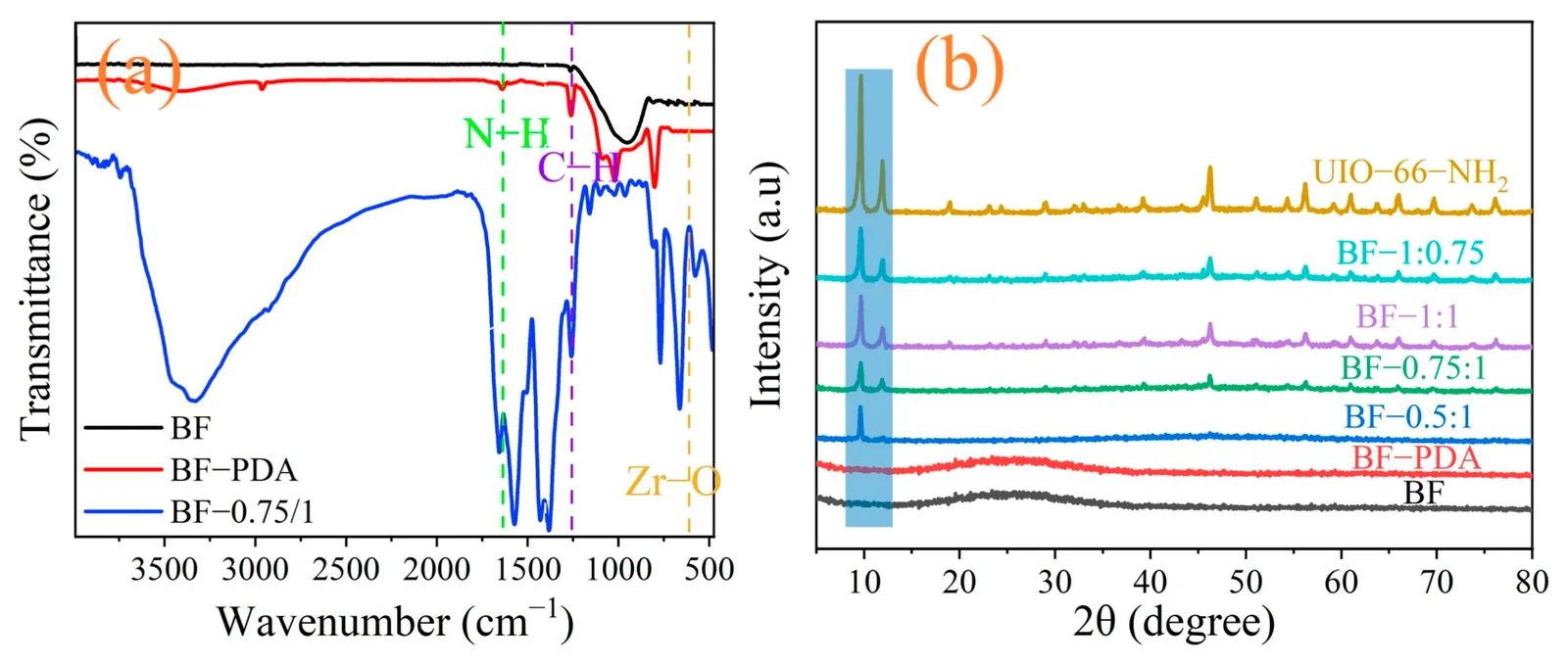
(**a**) Infrared spectra of BF before and after modification; (**b**) XRD patterns of BF before and after modification.

FTIR (Figure 8a) showed the characteristic BF bands near 2950 and 1650 cm^−1^, 1000–1100 and 460 cm^−1^, and 3400/1630 cm^−1^. After PDA deposition, the features at 1505 and 1280 cm^−1^ were assigned to N–H/aromatic C=C and C–N vibrations [8,26,28]. BF-PDA/U-0.75:1 additionally showed the NH2-BDC carboxylate bands at 1580 and 1390 cm^−1^ and Zr–O modes at 650–800 cm^−1^ [13]. XRD (Figure 8b) gave broad halos for BF and BF-PDA, The blue-shaded region in (Figure 8b) marks the characteristic diffraction range of UiO-66-NH_2_, which covers the diffraction peaks corresponding to the (111) and (200) crystal planes of UiO-66-NH_2_. while BF-PDA/U-0.75:1 showed reflections at 2θ = 7.4°, 8.6°, 12.1°, 14.2°, 17.1°, 22.6°, 26.0° and 33.2°, indexed to the (111), (200), (220), (311), (222), (400), (331) and (420) planes of UiO-66-NH_2_. The Scherrer size from (111) was ~62 nm, consistent with the SEM observation [27].

**Figure 9:**
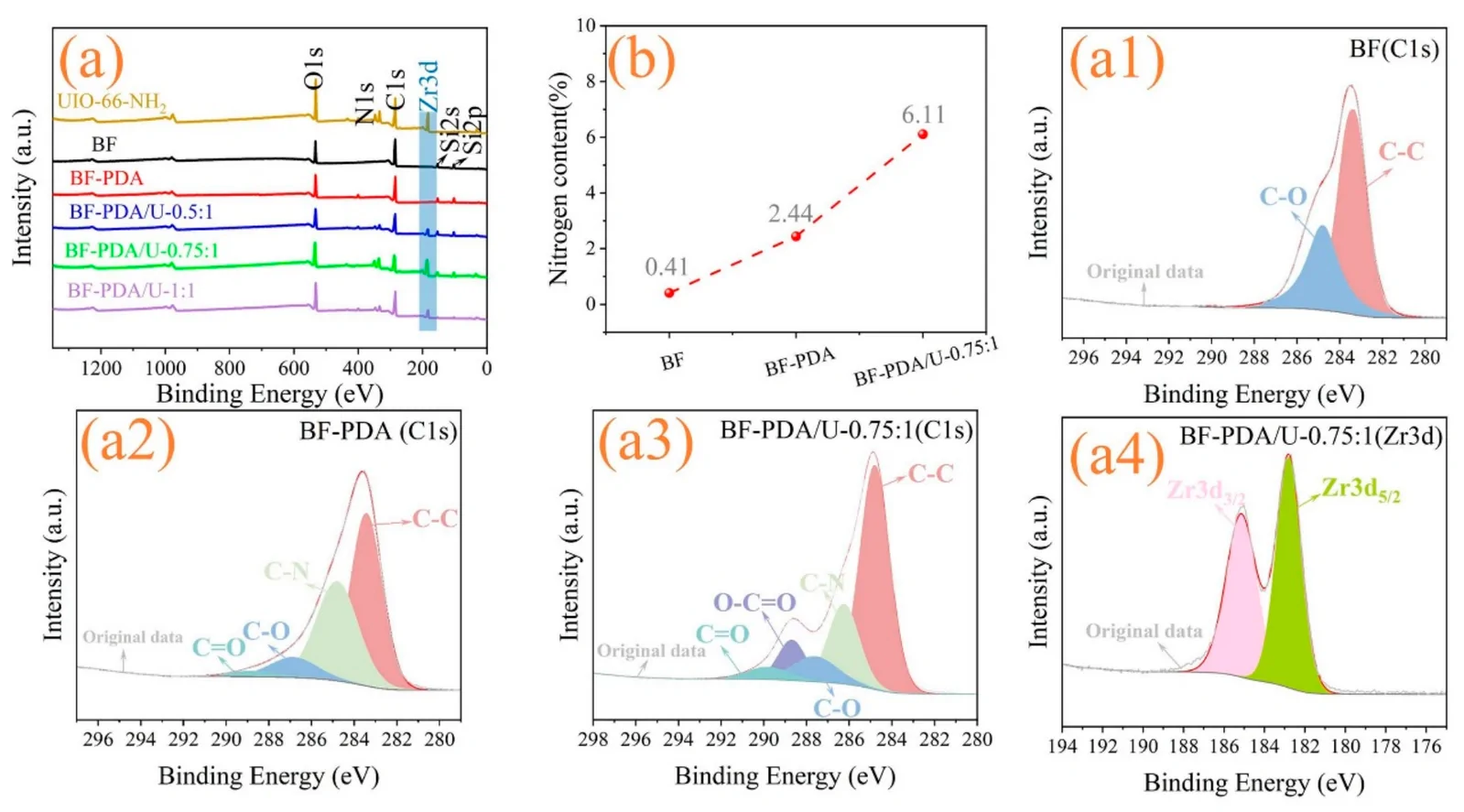
XPS survey spectra (**a**); N element content (**b**); high-resolution C1s spectra of BF (**a1**), BF-PDA (**a2**), and BF-PDA/U-0.75:1 (**a3**); and high-resolution Zr3d spectrum of BF-PDA/U-0.75:1 (**a4**).

XPS (Figure 9) tracked the surface chemistry. N rose from 0% for BF to 2.44% for BF-PDA and 6.11% for BF-PDA/U-0.75:1; the latter also showed a Zr 3d doublet. The BF C 1s components at 284.6, 285.8 and 287.9 eV correspond to C–C/C–H, C–O and O–C=O; PDA introduced a C–N/C=N contribution at 285.6–286.2 eV. For BF-PDA/U-0.75:1, enhanced aromatic/carboxylate contributions and Zr 3d_5_/2 at 182.5 eV (3d splitting 2.4 eV; 182.5/184.9 eV) support retention of the PDA interlayer and UiO-66-NH_2_ growth [10,26].

### 3.2. Monofilament Mechanical Properties of Bf Before and After Modification

#### 3.2.1. Monofilament Strength and Weibull Distribution of Fibers

**Figure 10:**
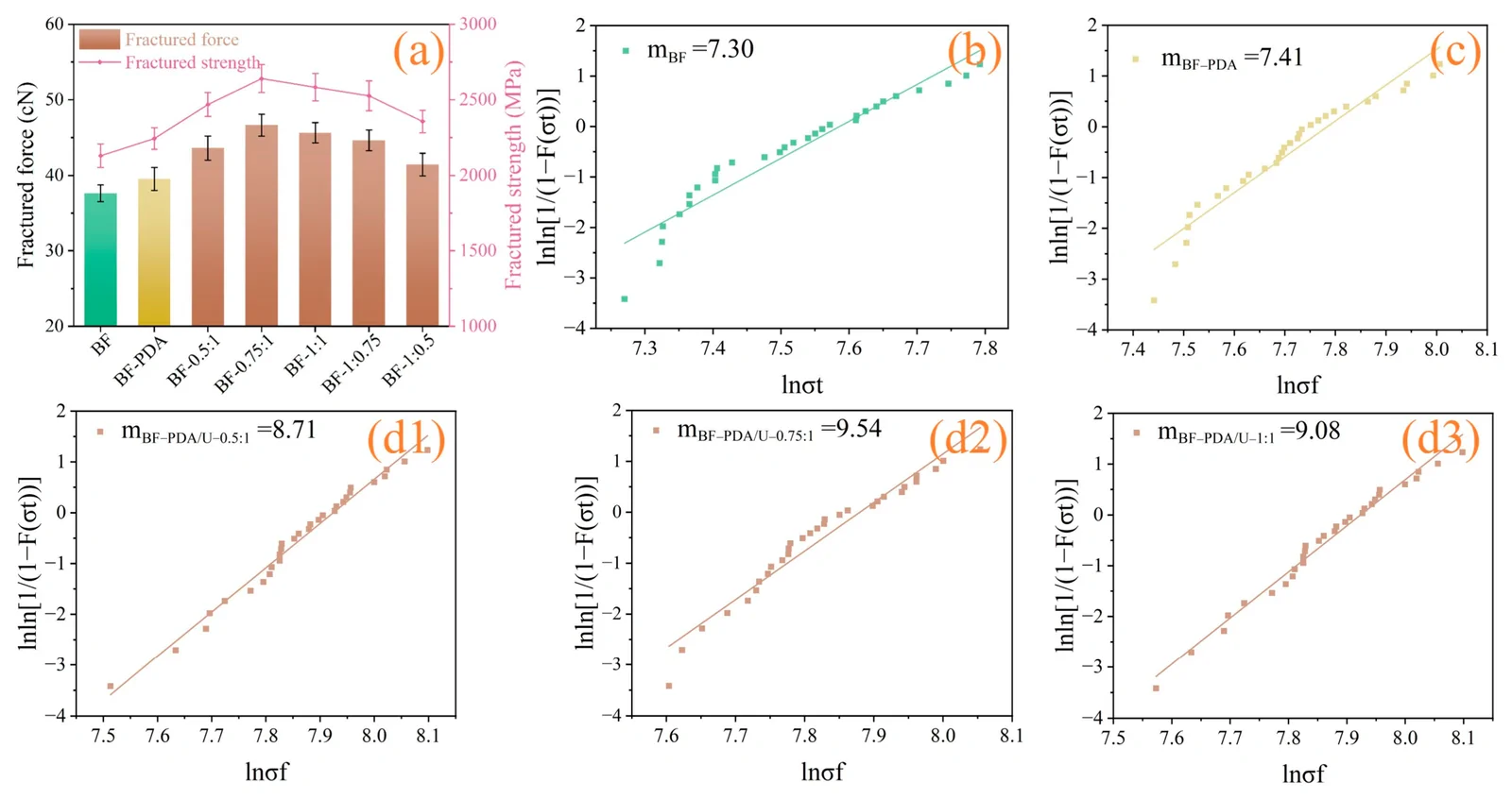
Performance characteristics of different BFs: (**a**) single-fiber tensile fracture strength; (**b**) Weibull distribution of BF; (**c**) Weibull distribution of BF-PDA; (**d1**–**d3**) Weibull distributions of BF-PDA/U-0.5:1, BF-PDA/U-0.75:1, and BF-PDA/U-1:1, respectively.

Single-fiber tests (*n* ≥ 50; Figure 10) gave 2.13 GPa for BF and 2.24 GPa (+5.16%), 2.46 GPa (+15.49%), 2.64 GPa (+23.94%), 2.58 GPa (+21.12%), 2.52 GPa (+18.30%) and 2.35 GPa (+10.32%) for BF-PDA, BF-PDA/U-0.5:1, BF-PDA/U-0.75:1, BF-PDA/U-1:1, BF-PDA/U-1:0.75 and BF-PDA/U-1:0.5, respectively. The Weibull modulus increased from 7.30 for BF to 7.41, 8.71, 9.54 and 9.08 for BF-PDA, BF-PDA/U-0.5:1, BF-PDA/U-0.75:1 and BF-PDA/U-1:1, respectively, indicating the narrowest measured distribution for BF-PDA/U-0.75:1 [29].

The trend is consistent with PDA covering surface defects and with a uniform, well-adhered MOF coating redistributing local stress; large or poorly distributed particles at non-optimal ratios can instead act as defects. The morphology and spectroscopy support this interpretation but do not directly demonstrate coordination, π–π interactions or particle-level load bearing [28,29,30].

#### 3.2.2. Resin Contact Angle and Interfacial Shear Strength of Fibers

**Figure 11:**
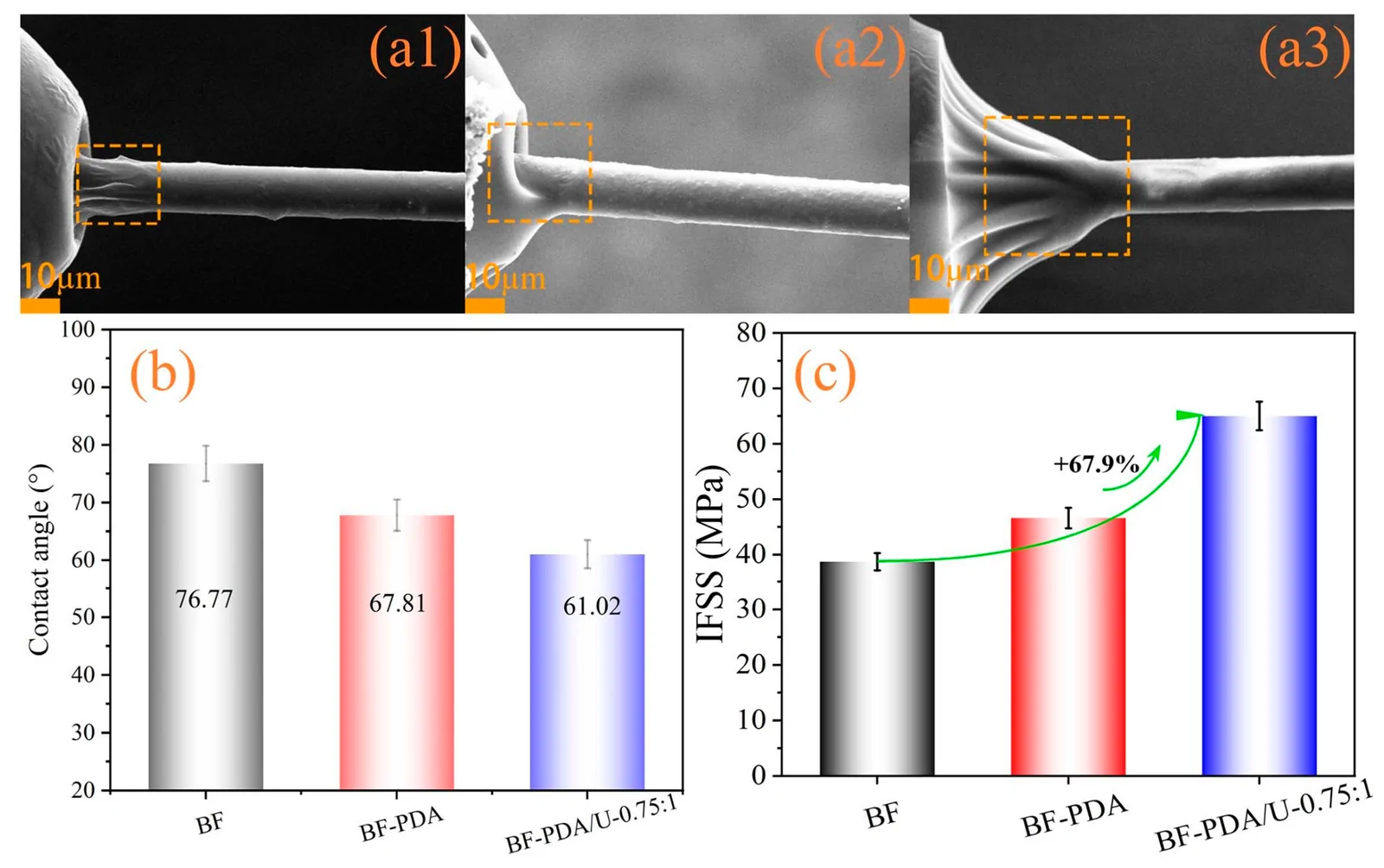
Performance characteristics of different BFs: (**a1**–**a3**) SEM images of microdroplet debonding surface morphology; (**b**) epoxy contact angle; (**c**) interfacial shear strength (IFSS).

The epoxy contact angle decreased in the order BF > BF-PDA > BF-PDA/U-0.75:1, reaching 61.02° ± 2.77° for the optimized fiber; water contact angles followed the same order (78.6° ± 3.1°, 66.4° ± 2.8° and 54.9° ± 2.4°). IFSS rose from 38.7 MPa for BF to 46.6 MPa for BF-PDA and 65.01 MPa for BF-PDA/U-0.75:1 (+67.9%; *n* ≥ 20 successful tests), while the Weibull modulus rose from 5.8 to 9.2 (Figure 11). Fractography changed from an almost bare BF surface to partial resin retention on BF-PDA and tightly adhered epoxy fragments on the optimized fiber, consistent with improved wetting and interfacial adhesion [27,29].

### 3.3. Mechanical Properties and Fracture Morphology of Bf/Ep Composites

**Figure 12:**
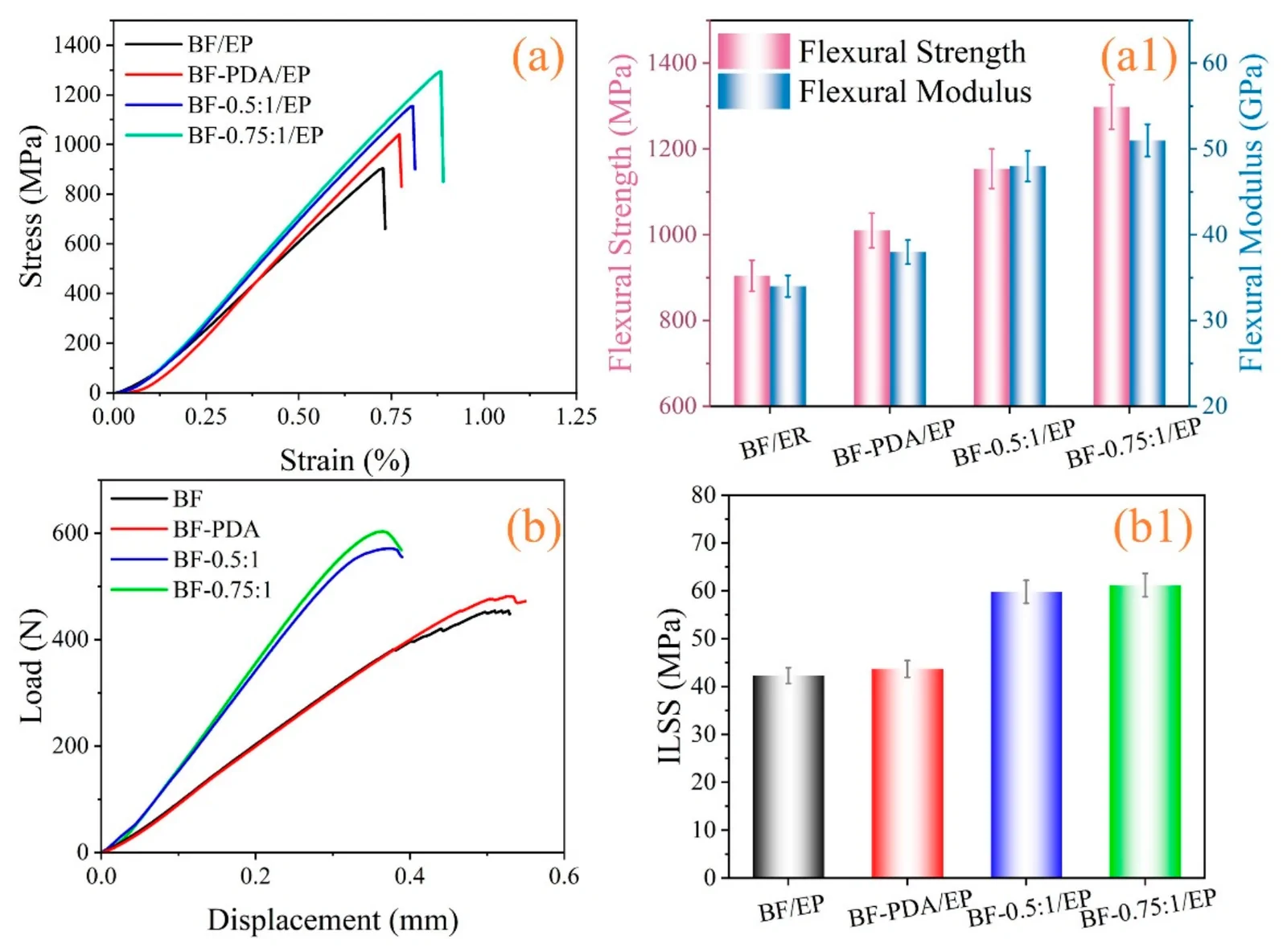
(**a**) Bending stress–strain curve, (**a1**) bending strength and bending modulus values, (**b**) typical force–displacement curve in ILSS and (**b1**) ILSS values.

Figure 12 summarizes laminate properties. Relative to those for BF/EP, the ILSS, flexural strength, and flexural modulus of BF-PDA/EP increased by 3.3%, 11.7%, and 11.8%, respectively. For BF-PDA/U-0.75:1/EP, the corresponding values reached 61.2 MPa, 1298 MPa, and 51 GPa, increases of 44.6%, 43.5%, and 49.2%. With the same fiber volume fraction (45 ± 2%) in all laminates, these gains are consistent with the improved wetting, IFSS, and hierarchical surface morphology [31,32]. Several modified specimens reached peak load at lower cross-head displacement, indicating a stiffer, slightly less ductile response [30,33].

**Figure 13:**
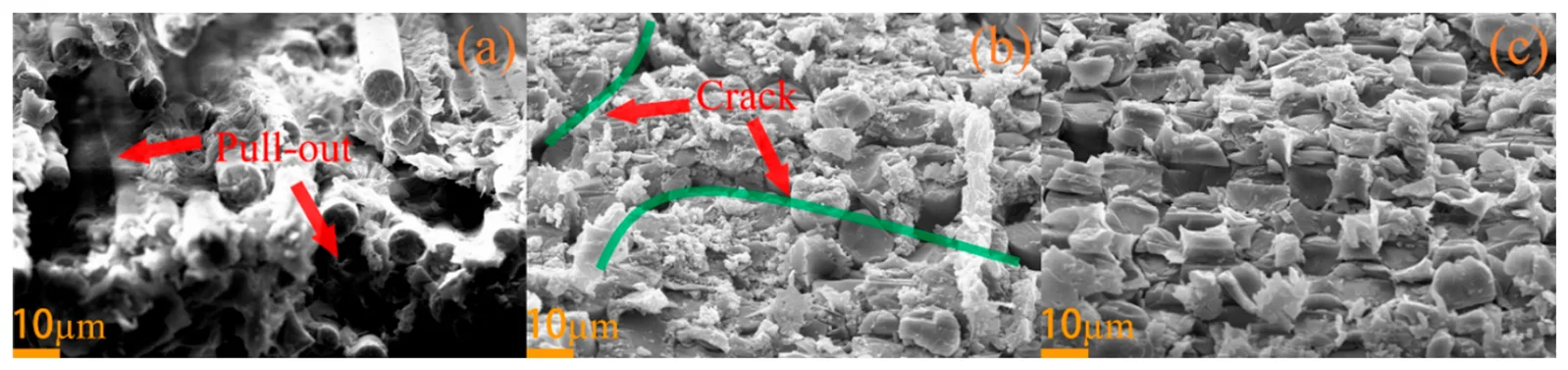
(**a**) BF/EP, (**b**) BF-PDA/EP, and (**c**) BF-PDA/U-0.75:1/EP cross-sectional SEM.

The fracture morphology (Figure 13) corroborated the interfacial trend. For unmodified BF/EP, massive fiber pull-out and interfacial voids manifested typical weak interfacial debonding failure. After PDA modification, large-scale debonding was suppressed, yet micro-cracks still existed. For BF-PDA/U-0.75:1/EP, uniform fiber fracture lengths and limited pull-out voids proved that failure mainly occurred inside the resin phase (cohesive failure), which is a signature of substantially enhanced fiber–matrix interfacial adhesion.

### 3.4. Mechanical Properties and Surface Morphology of Bf After Dynamic Seawater Corrosion

#### 3.4.1. Mechanical Properties of Bf After Dynamic Seawater Corrosion

**Figure 14:**
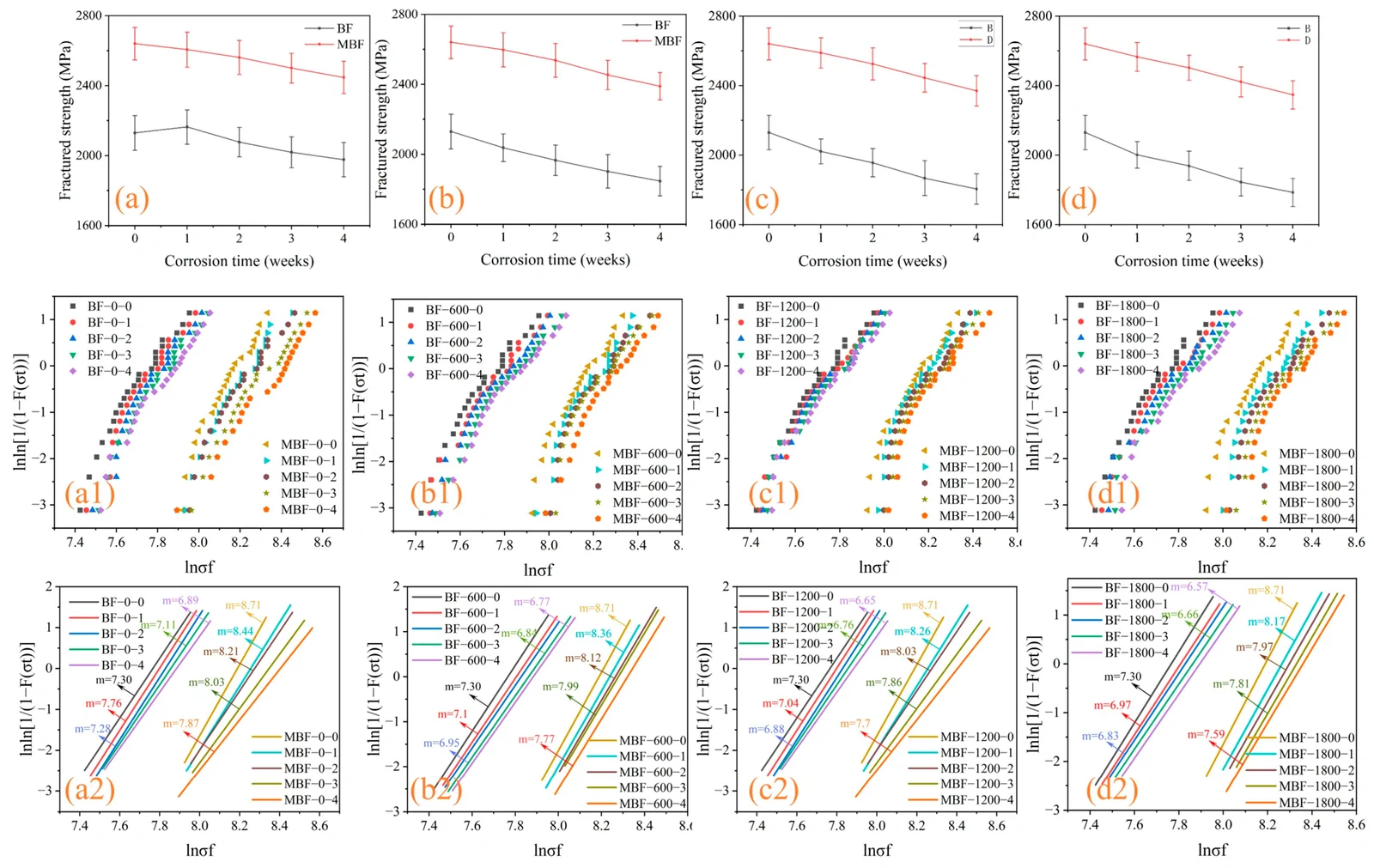
Changes in single-filament tensile strength at different speeds: (**a**) 0 rpm; (**b**) 600 rpm; (**c**) 1200 rpm; (**d**) 1800 rpm. Weibull distributions of single-filament tensile strength: (**a1**) 0 rpm; (**b1**) 600 rpm; (**c1**) 1200 rpm; (**d1**) 1800 rpm. Weibull moduli of single-filament tensile strength: (**a2**) 0 rpm; (**b2**) 600 rpm; (**c2**) 1200 rpm; (**d2**) 1800 rpm.

Figure 14 shows the monofilament tensile strength after dynamic seawater corrosion. The initial strengths were 2130 MPa for BF and 2640.5 MPa for modified BF (MBF). After 4 weeks, the BF and MBF strengths were, respectively, 1977 and 2446.7 MPa at 0 rpm (losses of 7.1% and 7.3%), 1847 and 2388.7 MPa at 600 rpm (13.2% and 9.5%), 1805 and 2369.89 MPa at 1200 rpm (15.2% and 10.2%), and 1785 and 2346.89 MPa at 1800 rpm (16.1% and 11.1%). The corresponding Weibull distributions are shown in Figure 14(a1–d2). MBF therefore retained a clear strength advantage under every corrosion condition.

Strength declined with the exposure time and stirring rate for both fiber types, while MBF consistently showed smaller losses. The surface and mass-change results are consistent with the hybrid coating limiting direct exposure of the fiber surface and moderating hydrodynamic damage [34,35].

#### 3.4.2. Surface Morphology of Bf After Dynamic Seawater Corrosion

**Figure 15:**
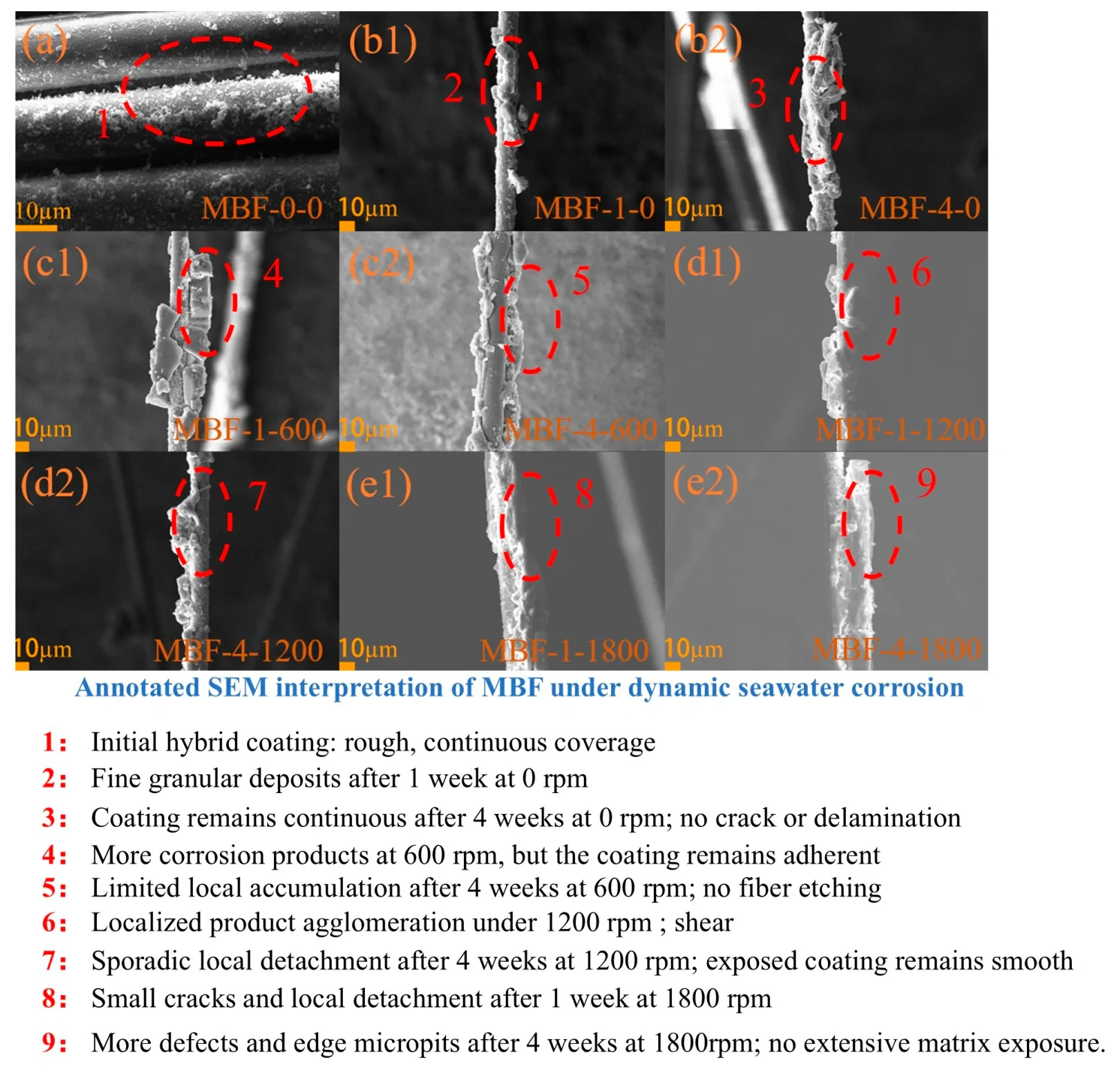
Surface morphology of different BFs under dynamic seawater corrosion at different speeds: (**a**) MBF-0-0; (**b1**) MBF-1-0; (**b2**) MBF-4-0; (**c1**) MBF-1-600; (**c2**) MBF-4-600; (**d1**) MBF-1-1200; (**d2**) MBF-4-1200; (**e1**) MBF-1-1800; (**e2**) MBF-4-1800.

Figure 15 compares BF and MBF after 1 and 4 weeks of dynamic corrosion under each stirring condition.

For unmodified BF, corrosion products changed from strip-like to block-like and sheet-like deposits with increasing stirring rate and exposure time. After 4 weeks at 1800 rpm, product spalling, etched pits, microcracks, and local fiber fracture were evident.

For MBF, the coating remained largely continuous at 0 and 600 rpm after 4 weeks, with limited local product accumulation and no obvious fiber etching. At 1200 rpm, local product removal and sporadic detachment were observed after 4 weeks. The most pronounced deterioration occurred at 1800 rpm, where the occurrence of small cracks, localized detachment, and a few edge micropits increased from 1 to 4 weeks; extensive matrix exposure, fracture, and large-area etching were not observed.

Compared with BF, the more continuous PDA/UiO-66-NH_2_ layer on MBF is consistent with a barrier to ion and water ingress and improved resistance to hydrodynamic removal. The general degradation behaviors of basalt fiber-reinforced composites in marine corrosive environments have been summarized in previous reports [36,37]. This interpretation agrees with the reduced mass change and the higher residual strength of the modified system.

### 3.5. Effect of Dynamic Seawater Corrosion on Bfrp Mass Change and Mechanical Properties

#### 3.5.1. Effect of Dynamic Seawater Corrosion on Bfrp Mass Change

**Figure 16:**
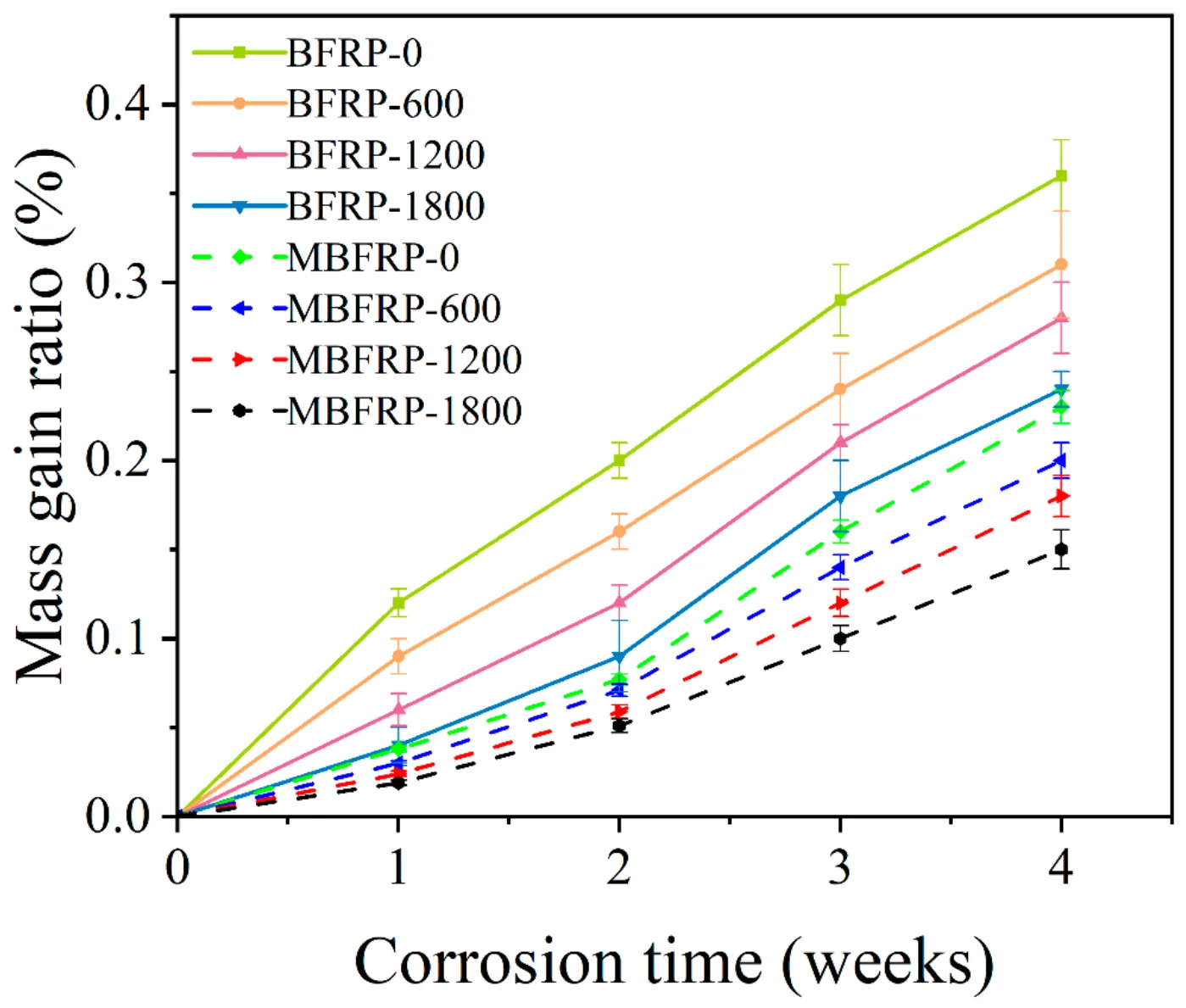
Mass changes of different BFRPs under different corrosion conditions.

Figure 16 shows the mass changes of unmodified BFRP and modified MBFRP under dynamic seawater exposure. Both materials gained mass overall, whereas the apparent diffusion coefficient D of MBFRP was lower than that of BFRP at all stirring rates.

MBFRP showed lower mass change under every condition. After 4 weeks, the BFRP mass gains were 0.24%, 0.28%, 0.31%, and 0.36% at 0, 600, 1200, and 1800 rpm, respectively; the corresponding MBFRP values were 0.15%, 0.18%, 0.20%, and 0.23%. This lower uptake is consistent with a more effective interphase barrier, particularly under more severe stirring.

#### 3.5.2. The Effect of Dynamic Seawater Corrosion on the Bending Strength of Bfrp

**Figure 17:**
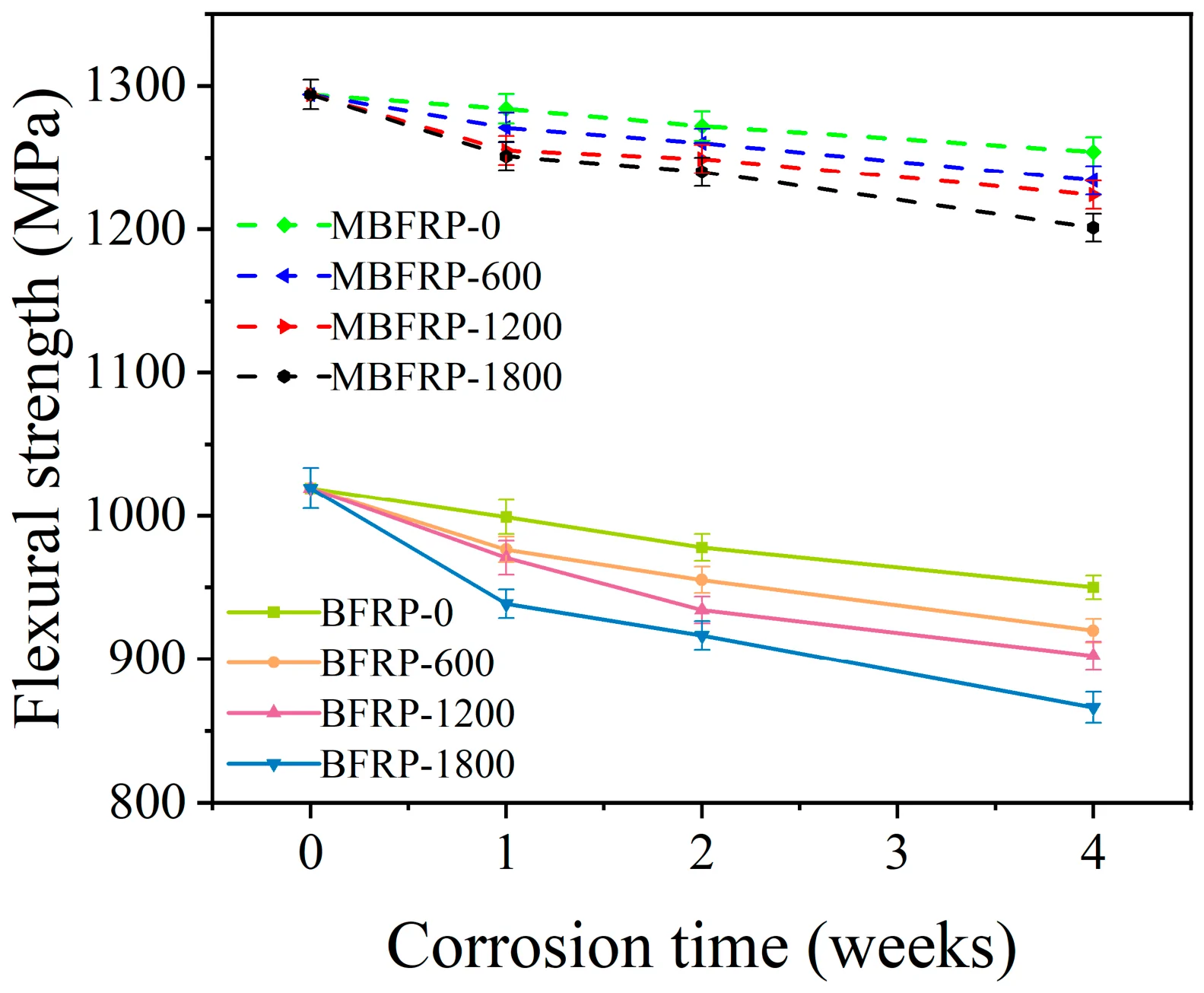
Variation in flexural strength of different BFRPs under different corrosion conditions.

Flexural strength decreased with immersion time under all conditions (Figure 17). After 4 weeks at 0, 600, 1200, and 1800 rpm, the BFRP strengths were 949.97, 919.82, 902.07, and 866.15 MPa (losses of 6.7%, 9.7%, 11.4%, and 15.0%), while the MBFRP strengths were 1254, 1234, 1224, and 1201 MPa (losses of 3.0%, 4.6%, 5.4%, and 7.1%). The flexural modulus followed the same ranking. At 1800 rpm, MBFRP retained 92.9% of its flexural strength, versus 85.0% for BFRP.

#### 3.5.3. The Effect of Dynamic Seawater Corrosion on the Shear Strength of Bfrp

**Figure 18:**
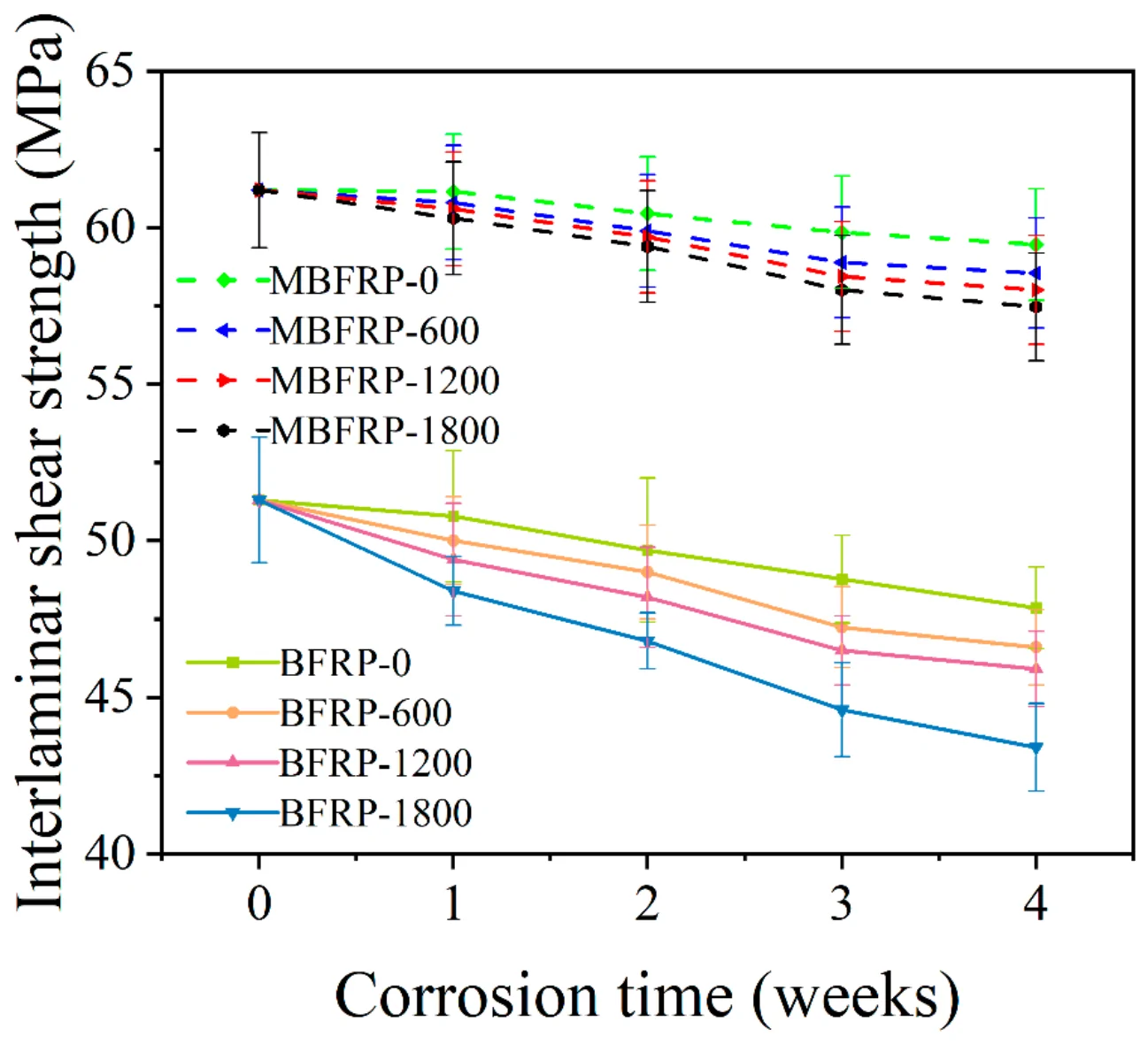
Shear strength variations of different BFRPs under different corrosion conditions.

Figure 18 shows the ILSS after exposure. After 4 weeks at 0, 600, 1200, and 1800 rpm, the BFRP ILSS values were 47.86, 46.60, 45.90, and 43.40 MPa (losses of 6.7%, 9.1%, 10.5%, and 15.3%), while the MBFRP values were 59.46, 58.55, 58.01, and 57.47 MPa (losses of 2.8%, 4.3%, 5.2%, and 6.0%). The lower degradation of MBFRP is consistent with its lower mass uptake and higher residual flexural strength [5].

### 3.6. Comparison with Reported Interface Modification Strategies

Table 2 compares the present interface and laminate properties with those of representative modified BF/epoxy systems. It is worth noting that MOF-modified carbon fiber composites have also been reported to achieve favorable interfacial and self-healing performance, providing an additional reference for MOF–fiber composite design [38]. The PDA/UiO-66-NH_2_ interface provided simultaneous increases in IFSS (+67.9%), ILSS (+44.6%), and flexural strength (+43.5%). Its durability was also quantified under dynamic seawater exposure: after 4 weeks at 1800 rpm, the modified laminate retained 92.9% of its flexural strength and 94.0% of its ILSS, compared with 85.0% and 84.7% for BFRP.

## 4. Conclusions

A PDA-assisted in situ growth strategy was developed to construct a PDA/UiO-66-NH_2_ hybrid interphase on basalt fibers. A 36 h PDA deposition time and a ZrCl_4_:NH_2_-BDC ratio of 0.75:1 produced a uniform, dense coating, as supported by SEM/EDS, FTIR, XRD, XPS, and AFM. The optimized modification increased the single-fiber tensile strength by 23.9% and IFSS by 67.9%; the laminate’s flexural strength and ILSS increased by 43.5% and 44.6%, respectively. It also reduced the apparent diffusion coefficient by 22–31% and lowered the mass change under all tested dynamic seawater conditions.

Under dynamic seawater exposure, the modified fibers and laminates showed lower mass variation and greater mechanical property retention than the unmodified counterparts. The results support the use of a hierarchical PDA/UiO-66-NH_2_ interphase to improve the load transfer and short-term seawater durability of BFRP composites. Long-term coupled environmental ageing and scale-up remain to be evaluated.

## Figures and Tables

**Table 1:** Concentrations of compounds in simulated seawater.

Solvent	Concentration (g/L)
NaCl	24.53
MgCl_2_	5.2
Na_2_SO_4_	4.09
CaCl_2_	1.16
KCl	0.695

**Table 2:** Quantitative comparison of interfacial and mechanical performance of modified basalt fiber/epoxy composites with representative literature data.

Fiber Treatment	System	Key Reported Property (MPa)	Improvement vs. Reference	Seawater Durability	Reference
Untreated (reference)	BF/epoxy	IFSS 38.7 ILSS 42.3 flexural 905		85.0% flexural/84.7% ILSS after-4-1800	This work
Tannic acid + APTES	BF/epoxy	Interfacial and mechanical improvement	Improved interfacial adhesion	Not evaluated	Ref. [39]
Dual tannic acid	BF/epoxy	Mechanical property improvement	Improved mechanical properties	Not evaluated	Ref. [40]
Bioactive cross-scale functionalization	BF composite insulation	Enhanced seawater corrosion resistance	Improved durability	Seawater	Ref. [4]
MOF-based oil containers (ex situ)	BF/epoxy	Mechanical and tribological improvement	Improved friction/wear performance	Not evaluated	Ref. [30]
MOF + PDA hybrid (carbon fiber)	CF/phenolic	Interfacial enhancement	Improved interfacial and tribological performance	Not evaluated	Ref. [16]
PDA + in situ UiO-66-NH_2_ (this work)	BF/epoxy	IFSS 65.01 ILSS 61.2 flexural 1298	IFSS + 67.9%; ILSS + 44.6%; flexural + 43.5%; D reduced by 22–31%	92.9% flexural/94.0% ILSS retention after 4 weeks, 1800 rpm	This work

## Data Availability

The original contributions presented in this study are included in the article/Supplementary Materials. Further inquiries can be directed to the corresponding author.

## Supplementary Materials

The following supporting information can be downloaded at: https://www.mdpi.com/article/10.3390/polym18192425/s1, Figure S1: Actual photos of three types of modified fibers; Figure S2: The preparation process of BFRP; Figure S3: The effect of PDA polymerization time on N elements; Figure S4: The effect of PDA polymerization time on Si elements; Figure S5: Stress-strain curves of BFRP after 4 weeks of corrosion at different speeds; Figure S6. Stress-strain curves of MBFRP after 4 weeks of corrosion at different speeds.
