# Supplementary material for: Enhanced Interfacial Adhesion and Dynamic Seawater Durability of Basalt Fiber/Epoxy Composites via PDA-Assisted In Situ Growth of UiO-66-NH2
Source: Polymers (Basel). 2026 Oct 5;18(19):2425. doi: 10.3390/polym18192425 (PMC13667093; doi:10.3390/polym18192425)
Supplement: Supplementary file 1 [file polymers-18-02425-s001.zip › polymers-4566556-supplementary.pdf]

Enhanced Interfacial Adhesion and Dynamic Seawater Durability of Basalt Fiber/Epoxy Composites via PDA-Assisted In Situ Growth of UiO-66-NH_2

Yingquan Zhou [1], Guoqian Xie [1], Bin Wang [1,2], Chunxia Zhao [1,2], Hui Li [1,2] and Dong Xiang [1,2,*]

1 School of New Energy and Materials, Southwest Petroleum University, Chengdu 610500, China;

18384914476@163.com (Y.Z.); 18331953657@163.com (G.X.); binwang2008@swpu.edu.cn (B.W.); polychem@swpu.edu.cn (C.Z.); lihui@swpu.edu.cn (H.L.)

2 Sichuan Engineering Technology Research Center of Basalt Fiber Composites Development and Application,

Southwest Petroleum University, Chengdu 610500, China

* Correspondence: d.xiang@swpu.edu.cn

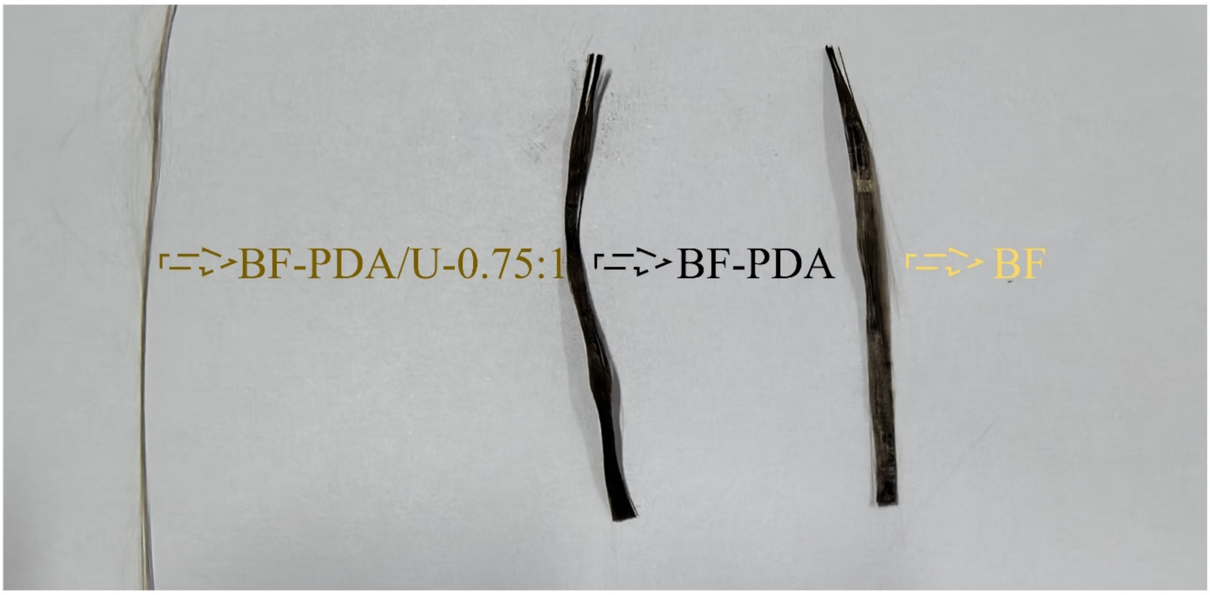

Figure S1. Actual photos of three types of modified fibers.

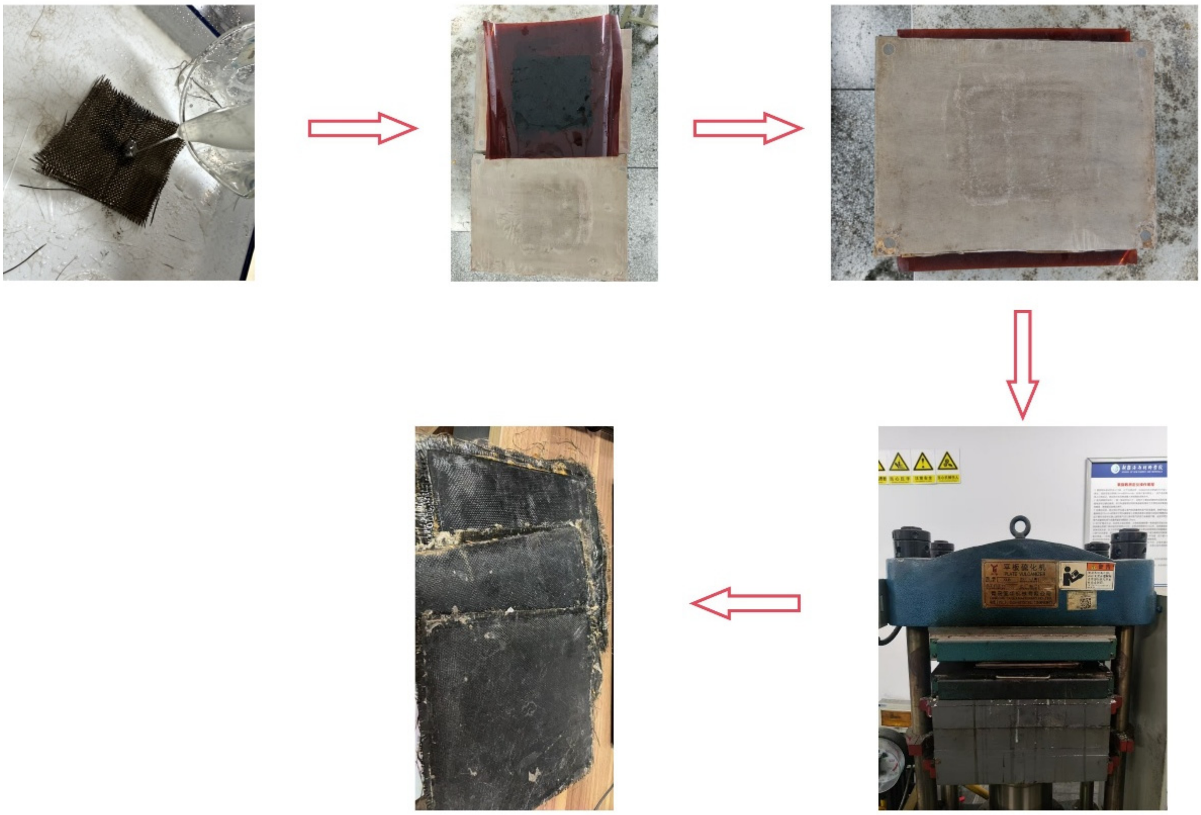

Figure S2. The preparation process of BFRP.

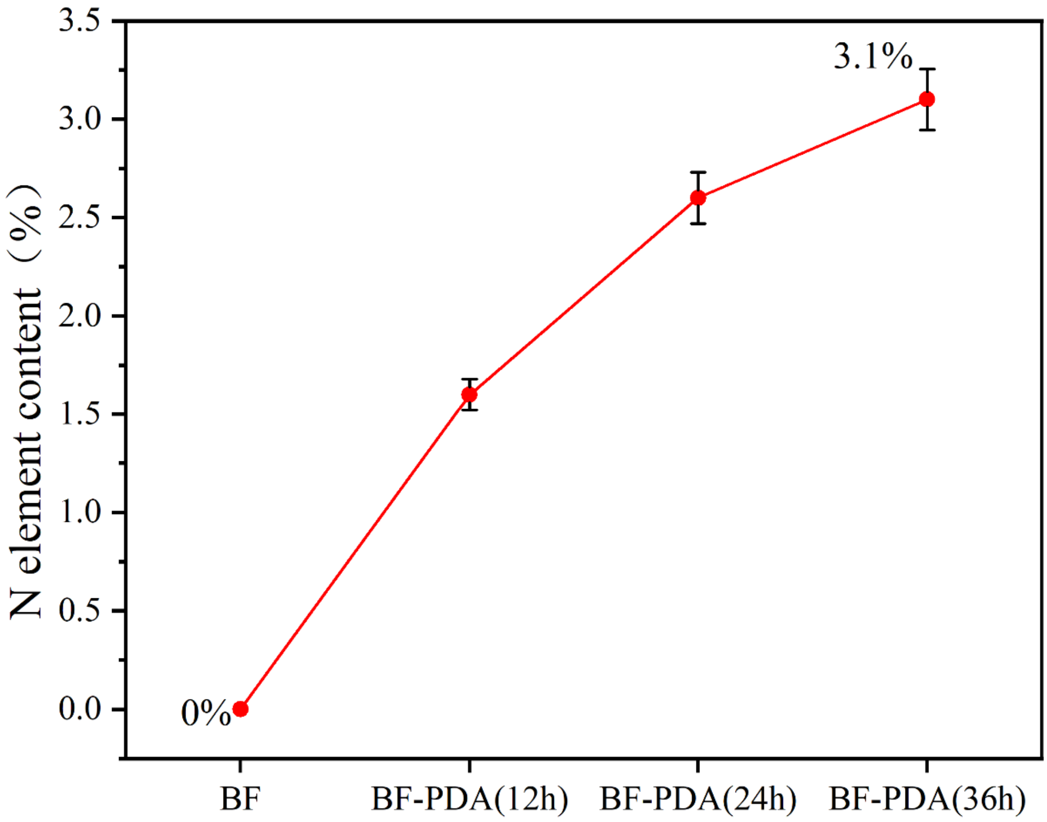

Figure S3. The effect of PDA polymerization time on N elements.

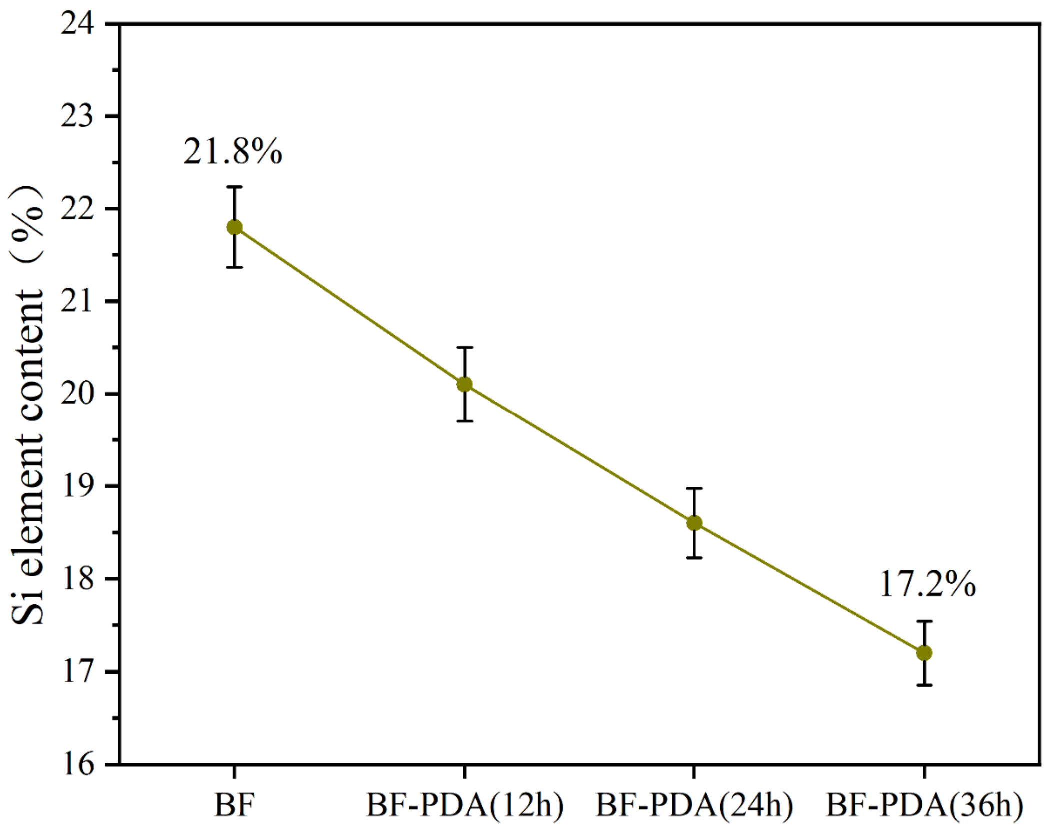

Figure S4. The effect of PDA polymerization time on Si elements.

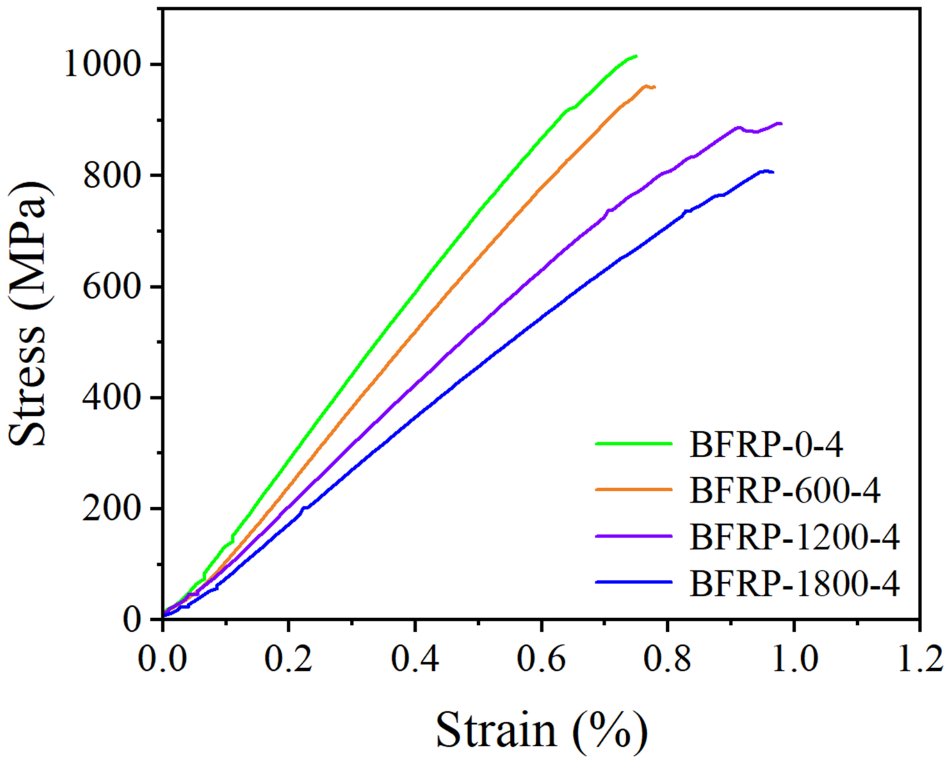

Figure S5. Stress-strain curves of BFRP after 4 weeks of corrosion at different speeds.

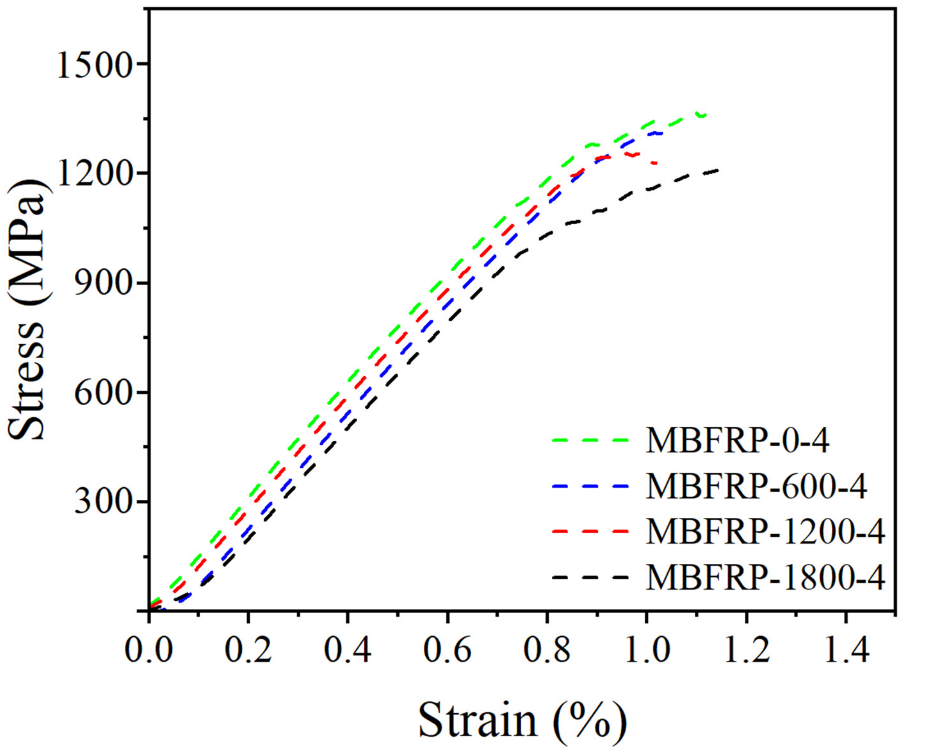

Figure S6. Stress-strain curves of MBFRP after 4 weeks of corrosion at different speeds.
